# Modular Nanoparticle Platform for Solution-Phase Optical Sensing of Protein–Protein Interactions

**DOI:** 10.1021/acsaom.4c00486

**Published:** 2025-03-15

**Authors:** Jieying Zhou, Korneel Ridderbeek, Peijian Zou, Aaron B. Naden, Stefan Gaussmann, Fangyuan Song, Pascal Falter-Braun, Euan R. Kay, Michael Sattler, Jian Cui

**Affiliations:** † Helmholtz Pioneer Campus, Helmholtz Munich, Neuherberg 85764, Germany; ‡ Institute of Structural Biology, Molecular Targets and Therapeutics Center, Helmholtz Munich, Neuherberg 85764, Germany; § Bavarian NMR Center, Department of Bioscience, School of Natural Sciences, Technical University of Munich, Garching 85748, Germany; ∥ EaStCHEM School of Chemistry, University of St. Andrews, St. Andrews KY16 9ST, U.K.; ⊥ Institute of Network Biology (INET), Molecular Targets and Therapeutics Center (MTTC), Helmholtz Munich, Neuherberg 85764 Germany; # Microbe-Host Interactions, Faculty of Biology, Ludwig-Maximilians-Universität (LMU) München, Planegg-Martinsried 82152, Germany; ∇ Department of Bioscience, School of Natural Sciences, Technical University of Munich, Garching 85748, Germany

**Keywords:** plasmonic nanoparticles, protein−protein interactions, optical biosensing, solution-phase sensing, surface functionalization, localized surface plasmon resonance, binding constant, binding kinetics

## Abstract

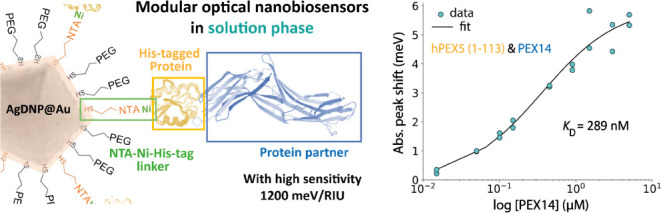

Protein–protein interactions regulate essentially all cellular processes. Understanding these interactions, including the quantification of binding parameters, is crucial for unraveling the molecular mechanisms underlying cellular pathways and, ultimately, their roles in cellular physiology and pathology. Current methods for measuring protein–protein interactions *in vitro* generally require amino acid conjugation of fluorescent tags, complex instrumentation, large amounts of purified protein, or measurement at extended surfaces. Here, we present an elegant nanoparticle-based platform for the optical detection of protein–protein interactions in the solution phase. We synthesized gold-coated silver decahedral nanoparticles possessing high chemical stability and exceptional optical sensing properties. The nanoparticle surface is then tailored for specific binding to commonly used polyhistidine tags of recombinant proteins. Sequential addition of proteins to the nanoparticle suspension results in spectral shifts of the localized surface plasmon resonance that can be monitored by conventional UV–vis spectrophotometry. With this approach, we demonstrate both the qualitative detection of specific protein–protein interactions and the quantification of equilibrium and kinetic binding parameters between small globular proteins. Requiring minimal protein quantities and basic laboratory equipment, this technique offers a simple, economical, and modular approach to characterizing protein–protein interactions, holds promise for broad use in future studies, and may serve as a template for future biosensing technologies.

## Introduction

Protein–protein interactions are fundamental to cellular pathways, including signaling, spatial cellular organization, metabolism, and reproduction.^1^ Understanding not only which proteins interact with one another, but also the stability and kinetics of these interactions, is essential for understanding the molecular mechanisms of cellular pathways, building accurate systems-level models of cells, and understanding events in physiology and the progression of disease.^2,3^ However, a key challenge remains the accurate and efficient measurement of the equilibrium binding affinities and kinetic parameters of protein–protein interactions.

At the systems level, proteomics methods such as mass spectrometry and yeast two-hybrid screening can detect protein–protein interactions with high throughput.^1,4,5^ However, these measurements are typically binary assessments—they can only measure whether an interaction has occurred or not within the measurement’s dynamic range. Therefore, *in vitro* measurements of purified proteins are widely used to characterize binding parameters, including the equilibrium dissociation constant (*K*_D_), and the kinetic rates of association (*k*_a_) and dissociation (*k*_d_).

These techniques span a wide array of modalities, each with its pros and cons concerning information provided, sensitivity, and sample requirements.^6^ The richest data are obtained from methods that do not perturb the protein sample. One widely used method is isothermal titration calorimetry (ITC),^7^ which can measure the dissociation constant (*K*_D_), thermodynamic parameters (enthalpy and entropy), and even binding stoichiometries, without any sample modification. However, it is a slow technique with limited temporal resolution and throughput while also requiring large amounts of protein sample. Nuclear magnetic resonance (NMR) can uniquely provide information on equilibrium and kinetic binding constants with residue-level structural information, providing in-depth insight into conformational changes or allosteric effects.^8,9^ However, it requires complex instrumentation and large amounts of purified protein, with some measurements also requiring NMR-active isotope labeling.

The high instrumentation and sample demands of NMR and ITC are due to a lack of sensitivity when measuring nuclear spins and heat. These demands can be overcome by introducing a modification to the system: a fluorescent probe, which serves as a high-contrast local reporter. Fluorescence-based techniques, such as fluorescence anisotropy,^10^ Förster resonance energy transfer (FRET),^11^ microscale thermophoresis,^12^ and stopped flow methods,^13^ can provide equilibrium and kinetic binding information with relatively simple instrumentation and modest sample amounts. However, conjugation of dyes to specific amino acids without perturbing protein binding can be nontrivial,^14,15^ often requiring modification of the protein sequence, thereby limiting the ease and throughput of these measurements.

Several methods avoid dye conjugation altogether *in lieu* of alternative readouts, often at the cost of immobilizing one binding partner.^16^ Surface plasmon resonance (SPR) and biolayer interferometry rely on perturbations of optical evanescent fields at solid–liquid interfaces to monitor protein binding.^17,18^ A relatively new technique based on DNA nanolevers monitors changes in the movement of DNA on metal surfaces upon protein binding.^19^ Among these techniques, SPR has emerged as the industry standard due to its ability to quantify kinetics rapidly, with small amounts of sample and without dye conjugation. However, downsides of SPR include high cost, complex instrumentation due to the integration of microfluidics and specialized metallic chips, and potential measurement artifacts related to restricted diffusion at the liquid–solid interface.^20−23^

A simpler and more economical alternative to SPR is the use of localized surface plasmon resonances (LSPRs) of colloidal metallic nanoparticles diffusing freely in solution.^24^ Here, biomolecular interactions at the LSPR “hotspots” of plasmonic nanoparticle suspensions produce spectral shifts that can be detected with conventional UV–vis spectrophotometry. Despite its innovative design and ease of use, this technique has remained underutilized, possibly due to its perceived limitation to the somewhat niche area of lipid–protein interactions.

In this study, we significantly advance this underexplored solution-phase LSPR concept by introducing a nanosensor design that extends its applicability to a domain of broader interest: protein–protein interactions. We first synthesized silver decahedral nanoparticles, an understudied class of plasmonic colloidal nanoparticles, possessing excellent optical sensing properties, including refractive-index sensitivity and optical sensing figure of merit (FOM). These nanoparticles were then coated with a thin layer of gold, improving chemical stability and compatibility with biological buffers while largely maintaining their optical sensing performance. Without such a coating, the silver decahedral nanoparticles would rapidly lose their sensing capabilities. We then chemically tailored the nanoparticle surface to reduce nonspecific protein interactions and also enable specific and modular immobilization of one protein binding partner via the polyhistidine tag of recombinant proteins. Finally, we demonstrated not only the qualitative detection of specific protein–protein interactions but also the quantification of binding parameters of proteins as small as ∼7 kDa, with good agreement with literature values. At the cost of immobilizing one protein binding partner, this technique offers a straightforward, low-cost, and modular approach for measuring protein–protein interactions without complex instrumentation and within the solution phase. These attributes make this technique an attractive method for studying the important problem of protein–protein interactions, with the potential to serve as a foundation for future solution-phase biomolecular sensing applications.

## Methods

### Silver Decahedral Nanoparticle (AgDNP) Synthesis

Silver decahedral nanoparticles (AgDNP) were synthesized by generally following a published protocol.^31^ In brief, a home-built photoreactor with 455 nm emission LEDs and a water-cooling system was used. Fourteen milliliters of Milli-Q water, 0.52 mL of 50 mM sodium citrate, 0.023 mL of 2 mg/mL PVP-40K, 0.025 mL of 5 mM l-Arginine, 0.4 mL of 5 mM AgNO_3_, and 0.2 mL of freshly prepared 0.1 M NaBH_4_ were added to a 20 mL glass scintillation vial. This solution was “aged” for 50 min in the dark, after which a bright yellow solution was formed. The vial was then positioned 10 mm above the LED of the photoreactor, and the entire photoreactor was shaken at 250 rpm for 10 min in the dark. Afterward, the LED was turned on (140 mW directly above the LED), and 0.3 mL 30% H_2_O_2_ was added while the setup was shaking. Shaking was maintained for 30 min and then stopped. Vials were illuminated for 14.5 h.

### Gold Coating of Silver Decahedral Nanoparticles (AgDNP@Au)

Gold coating of silver decahedral nanoparticles was based on a published protocol.^32^ In brief, 3 mL of a 0.0128 mM HAuCl_4_ aqueous solution was added to a 3 mL suspension of AgDNP at a rate of 0.25 mL/h over 12 h under 200 rpm stirring at room temperature in the dark. The resulting nanoparticles are the AgDNP@Au.

### Nanoparticle Characterization

Extinction spectra were measured using a V-760 UV–vis spectrophotometer (Jasco). Dynamic light scattering and zeta potential were measured on a Zetasizer Ultra (Malvern), using the multi angle dynamic light scattering (MADLS) mode for the size measurements and ZS XPLORER software for analysis. TEM images were collected with a Libra 120 instrument (Zeiss) operating at 120 kV. Nanoparticle samples were prepared on copper grids with lacey carbon films (Agar Scientific and Electron Microscopy Sciences). STEM was performed on a Titan Themis (FEI) operated at 200 kV and equipped with a DCOR probe corrector (CEOS), a SuperX energy dispersive X-ray spectrometer (EDX), and a Gatan Enfinium electron energy loss spectrometer (EELS). High-angle annular dark field (HAADF) images were acquired with a probe convergence angle of 21.2 mrad and inner/outer collection angles of 74 and 200 mrad, respectively. EELS spectra were acquired with a collection angle of 8.1 mrad; the background was subtracted using a power-law background, and the spectra were corrected for plural scattering by Fourier ratio deconvolution.

### Preparation of PEG/NTA-Ni-Modified AgDNP@Au “Nanobiosensors”

AgDNP@Au, after synthesis, were redispersed in Milli-Q water following centrifugation at 10870 g for 10 min. For each synthesis, 350 μL of nanoparticles, diluted to a peak extinction of 1.16 as measured by UV–vis spectroscopy, were placed in a 1.5 mL Eppendorf tube. A total amount of 375 μM ligand mixture containing mPEG-SH (MW 800, “PEG800” or MW 2000, “PEG2000”) and thiolated alkane-PEG-nitrilotriacetic acid ("NTA") was added for reaction at room temperature overnight under 250 rpm shaking. After separation from the reaction mixture by centrifugation at 10870 g for 10 min, the PEG/NTA-modified nanoparticles were washed twice with 0.1 M Tris buffer containing 0.025 wt % Tween 20. The particles were then incubated in 50 μM NiCl_2_ for 2.5 h at room temperature under 250 rpm shaking. Afterward, the particles were washed once with 20 mM Tris buffer containing 0.005 wt % Tween 20.

### Protein–Protein Interaction Sensing

The nanobiosensors were redispersed in different saline buffers in bovine serum albumin (BSA)-precoated cuvettes for protein sensing experiments. BSA precoating was performed using a 2.5% (w/v) BSA in PBS buffer, shaken thoroughly to touch all surfaces of the cuvettes for 20 min before removal. The coated surfaces were then washed with Tris-buffered saline and Milli-Q H_2_O, and dried using compressed air.

Desired amounts of protein solution were added to a given nanoparticle suspension. For measurements near equilibrium, the extinction spectrum (350–750 nm, 0.2 nm intervals) was monitored until no further peak shift was observable. A shaking speed of 250 rpm was applied between measurements to facilitate mixing.

For kinetic measurements, a smaller range of the extinction spectrum (±10 nm from the peak position, λ_max_) was scanned at a 0.1 nm interval. Once the second protein was added, the sample was measured every 20–30 s per scan for 25 min. After the kinetics measurement, the entire spectrum (350–750 nm, 0.2 nm interval) was scanned again to obtain the “final” spectrum.

### Protein Cloning and Purification

DNA sequences for TAD and NCBD were optimized according to the codon usage of*Escherichia coli (E. coli)* and synthesized by Integrated DNA Technologies (Europe). The genes were cloned into the pETM10 vector with a noncleavable N-terminal His_6_-tag, and the pETM11 vector with His_6_-tag followed by a tobacco etch virus (TEV) cleavage site (EMBL, G. Stier), respectively, using NcoI and *Kpn*I restriction sites. PEX5 (1–113), PEX5 (110–230), and PEX14 (16–80) constructs were obtained from previous work.^60^ The His-tagged protein samples were made from the proteins expressed with the pETM10 vector, and the non-His-tagged versions of the proteins were obtained from pETM11 constructs by following a TEV protease cleavage to remove the His-tag.

The constructs were transformed into (*E. coli*) BL21 (DE3) cells and expressed in lysogeny broth (LB) medium. A single colony was picked randomly and cultured in the medium with 50 μg/mL kanamycin overnight at 37 °C. Overnight cultures were grown in the medium at 37 °C, diluted 50-fold, and grown until an optical density of 0.4–0.6 at 600 nm was reached. Then, protein expression was induced by adding 0.5 mM Isopropyl β-D-1-thiogalactopyranoside (IPTG). The cultures were continuously incubated at 37 °C for 4 h and then switched to 25 °C for another 20 h. The cells were harvested by centrifugation at 5000 rpm for 20 min at 4 °C. Cell pellets were resuspended in the Ni-NTA binding buffer (30 mM Tris/HCl, pH 8.0, 300 mM NaCl, 10 mM imidazole, 1 mM TCEP) with the addition of 200 μg/mL lysozyme and 10 μg/mL DNase and lysed by pulsed sonication (3 min, 30% power, large probe, Bandelin UW 2200). The lysates were incubated at 4 °C for 20 min to digest chromosomes, followed by the addition of solid urea to the concentration of 4 M, a second sonication step as described above, and then centrifugation at 14000 rpm for 60 min at 4 °C. All proteins were purified using gravity flow Ni-NTA affinity chromatography (Qiagen) using 300 mM imidazole. Non-His-tagged proteins were further purified after TEV cleavage with a reverse Ni^2+^ column. All proteins were then purified by size exclusion chromatography (Superdex S75, 16/600, GE) in the buffer (25 mM HEPES, pH 7.5, 150 mM NaCl, and 1 mM TCEP).

## Results and Discussion

### Design, Synthesis, and Characterization of Nanoparticle Optical Biosensors

Nanoparticle optical sensors must satisfy several key criteria in order to sense protein–protein interactions in the solution phase. The first set of criteria is related to the physical properties of the nanoparticle: small size, high chemical and colloidal stability, and excellent optical sensing performance. The second set of criteria relates to how these nanoparticles interface with proteins: the nanoparticle surface should permit specific protein–protein interactions and eliminate nonspecific interactions, all while maintaining colloidal stability in biological buffers.

LSPR-based sensors respond optically to refractive-index changes within plasmonic “hotspots” located at the nanoparticle surface, typically at edges and tips. Given the small size of most proteins and their small refractive-index mismatch with water (∼1.45 vs. 1.33),^25^ the optical sensing performance of the nanoparticle is perhaps the most important criterion for protein detection. This performance is commonly encapsulated by the optical sensing figure-of-merit (FOM), which is the ratio of the nanoparticle’s refractive-index sensitivity to its spectral line width. In other words, the FOM is maximized with high refractive-index sensitivity and narrow spectral line width.

Both the refractive-index sensitivity and FOM are determined by the nanoparticle material composition, geometry, and size. Among plasmonic nanoparticles at visible wavelengths, silver (Ag) nanoparticles generally possess the highest refractive-index sensitivities and narrowest spectral line widths.^26−29^ Among known geometries, we identified silver decahedral nanoparticles (AgDNP)^30^ as a promising candidate for high-sensitivity refractive-index sensing. However, the low chemical stability of silver nanostructures in biological solutions prompted us to explore a gold (Au) coating, which imbues these nanoparticles with chemical stability, biocompatibility, and possibilities for surface functionalization.

### Synthesis of Ag Decahedral Nanoparticles with Thin Au Coating

**Figure 1:**
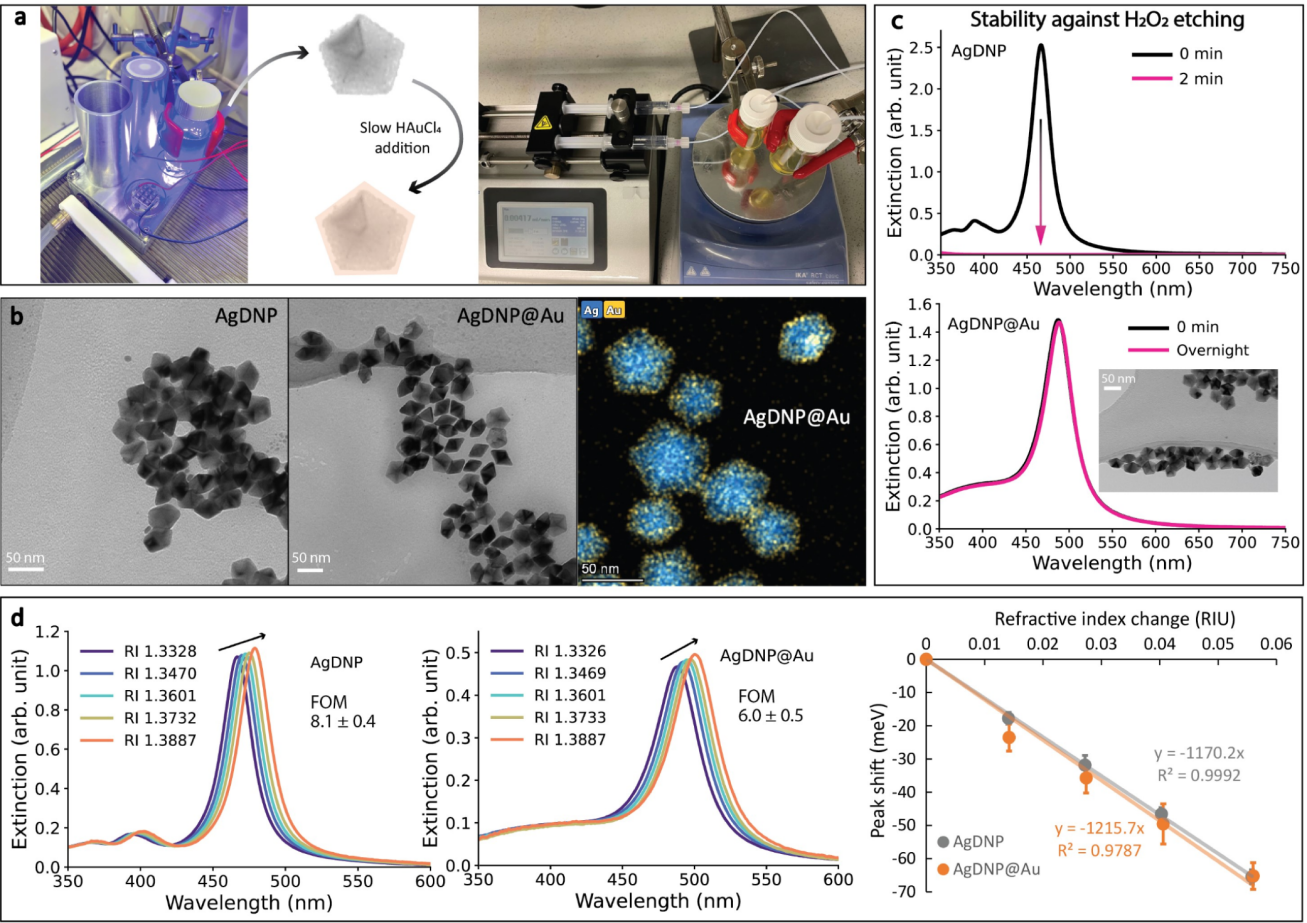
Synthesis and characterization of Ag decahedral nanoparticles (AgDNP) and AgDNP with Au coating. (a) Left: AgDNPs were synthesized in a home-built photoreactor (455 nm irradiation) equipped with a cooling system to minimize adverse heating effects. Up to six batches can be synthesized simultaneously. Right: A syringe pump was used to slowly inject a HAuCl_4_ solution over 12 h to form a thin Au coating on the AgDNPs. Up to two batches could be synthesized simultaneously. (b) TEM images of AgDNP (left) and Au-coated AgDNP (middle). STEM-EDX image of AgDNP@Au with Ag (blue) and Au (yellow) channels overlaid (right). (c) Stability test of AgDNP (top) and AgDNP@Au (bottom) against 1.0 M H_2_O_2_ etching. The TEM image inset shows AgDNP@Au after H_2_O_2_ etching overnight. (d) Refractive-index (RI) sensitivity spectra of AgDNP (left) and AgDNP@Au (middle) in glycerol–water solutions, with black arrows indicating the increasing RI. RI values were confirmed with a refractometer. In measurements of AgDNP, 2 mM sodium citrate was included in the solution to improve nanoparticle stability (see Figure S3). The spectral amplitude was corrected for dilution. Right: Linear fitting of spectral peak shifts vs RI change was used to derive RI sensitivities (RIU/meV).

We synthesized silver decahedral nanoparticles (“AgDNP”, ∼ 40 nm) under 455 nm irradiation^31^ using a custom-built, water-cooled photoreactor, as shown in Figure 1a . To improve the chemical stability and biocompatibility, a layer of Au was coated on the silver core by gradually adding HAuCl_4_ to the reaction mixture, resulting in so-called “AgDNP@Au”. The HAuCl_4_ solution was slowly applied over 12 h (Au:Ag = 1:10 molar ratio) in order to suppress the formation of hollow structures caused by galvanic replacement.^32^

We confirmed the presence of the Au coating by several methods. First, transmission electron microscopy (TEM) showed that the average lengths of the pentagon plane of the AgDNP and AgDNP@Au were 38.4 and 41.0 nm, respectively (Figure 1b; for additional images and size distribution analysis, see Figure S1). This slight difference was also reflected in dynamic light scattering (DLS), which showed hydrodynamic diameters of ∼38.7 and ∼42.5 nm for the particles before and after Au coating, respectively. Finally, the thickness of the Au layer was estimated to be roughly 1 nm, or 3–4 atomic layers,^33^ by scanning TEM (STEM) and energy-dispersive X-ray (EDX) elemental analysis (Figure S2).

Optically, the extinction spectra of the Ag decahedral nanoparticles showed a sharp peak around 472 nm with a full-width at half-maximum (fwhm) of 140 meV attributed to the longitudinal dipole LSPR mode (Figure 1c,d).^30^ The AgDNP@Au nanoparticles present a significantly red-shifted LSPR band around 492 nm with a broadened fwhm of 228 meV, presumably due to higher plasmonic damping of Au.^26,27,34^ We also observed that the transverse dipolar LSPR mode of AgDNP around 401 nm was significantly diminished after Au coating (Figure 1c,d).^30^ Next, we demonstrate how the ultrathin Au layer improves the chemical and colloidal stability of the nanoparticles without considerably hampering the optical-sensing properties of the Ag core.

### Stability of Au-Coated Ag Decahedral Nanoparticles

Nonspherical Ag nanoparticles are known to be chemically unstable as the release of Ag atoms from the high-energy facets occurs even under mild conditions.^32,33,35^ In an oxidative environment, nanoparticles can be etched away within minutes.^33,35^Figure 1c shows the stability of AgDNP and AgDNP@Au in a 1.0 M (3%) H_2_O_2_ solution before and after Au coating (for detailed etching procedures, see Supporting Information). The LSPR band of uncoated AgDNP vanished nearly instantly after the H_2_O_2_ addition. In contrast, the extinction spectrum of AgDNP@Au remained nearly unchanged after overnight exposure, with the large majority of nanoparticles remaining intact, as also seen in TEM (inset). These results suggest that the ultrathin Au coating coverage was not only essentially complete around the AgDNP cores but also sufficiently thick to protect the cores from oxidative reactions.

We further examined the stability of AgDNP and AgDNP@Au in commonly used biological buffers, including phosphate-buffered saline (PBS), HEPES-, and Tris-buffered saline buffers (for detailed procedures, see Supporting Information). We found that, after Au coating, the nanoparticle colloidal stability was substantially improved up to 24 h in all tested buffers (Figure S4). A reduced, but largely adequate, stability was observed at the physiological temperature of 37 °C up to 6 h (Figure S5). Altogether, the ultrathin Au shell evidently enhanced the chemical and colloidal stabilities of these nanoparticles, enabling the possibility of their use in biomolecular sensing.

### Refractive-Index Sensitivity and Figure-of-Merit (FOM) of Au-Coated Ag Decahedral Nanoparticles

The sensing ability of LSPR optical sensors is typically quantified with two metrics: the refractive-index (RI) sensitivity and the so-called figure-of-merit (FOM), which is the ratio of the RI sensitivity to the LSPR spectral line width. A large FOM is highly desired because a smaller perturbation, such as the presence of a protein, results in a more evident spectral shift.^26,36^ We determined the RI sensitivity and FOM of our AgDNP and AgDNP@Au by measuring their spectral shifts in varying glycerol–water mixtures, with the refractive index verified with a refractometer (for detailed procedures, see Supporting Information). Figure 1d shows the changes in the LSPR spectra of both Ag and Au-coated nanoparticles with increasing medium refractive index. As expected, the spectra red-shifted, broadened, and increased in intensity with increasing refractive index.^37^ The magnitudes of the spectral shifts were used to calculate the RI sensitivity.

Together with reported literature values, the RI-sensitivity and FOM values are summarized in Table 1. Our homemade 40 nm Ag decahedral nanoparticles possessed a longitudinal SPR mode at 472 nm, along with one of the highest-reported RI sensitivities of any nanoparticle at 1170 ± 51 meV/RIU. This value approaches some of the highest reported sensitivities for colloidal nanoparticles, such as 88 × 24 nm^2^ Ag triangular prisms^38^ and 84 nm^3^ Ag nanocubes,^26^ despite a much smaller physical size (Table 1). More strikingly, our AgDNP reached an FOM of 8.1 ± 0.4, nearly seven times the FOM of 84 nm^3^ Ag nanocubes,^26^ in large part due to the difference in spectral line width. This value is, by far, the highest FOM of any reported nanoparticle sensor to our knowledge.

**Table 1:** RI Sensitivity and FOM Values of Ag and Au Nanosensors in the Literature and in this Work^a^

Nanoparticles	Size (nm)	RI sensitivity (meV/RIU)	FOM	Reference
Au rod	73 × 41	640	2.1	(26)
Au cube	77	580	1.5	(26)
Au sphere	15	200	0.6	(39)
Au bipyramid	27 × 19	450	1.7	(39)
Ag sphere	40	1100	2.6	(40)
Ag triangular prism	88 × 24	1200	4.6	(38)
Ag cube	84	1400	1.2	(26)
Ag cube^b^	84	1000	4.6	(26)
Ag@SiO_2_	106	750	1.7	(24)
Au@Ag rod	49 × 25	900	3.1	(26)
**AgDNP**	**38**	**1200**	**8.1**	**This work**
**AgDNP@Au**	**41**	**1200**	**6.0**	**This work**

a Values have been rounded to two significant figures to account for potential measurement uncertainties.

b Quadrupole resonance.

Unexpectedly, we found that gold overcoating did not significantly reduce the RI sensitivity of AgDNP@Au (1216 ± 111 meV/RIU) compared to AgDNP (Figure 1d, right). This is a rather surprising finding, as for metallic nanoparticles with similar shape, size, and LSPR wavelength, the smaller real part of the dielectric function and the stronger plasmonic damping of Au compared to Ag should lead to a larger RI sensitivity of Ag nanoparticles.^26,27^ We tentatively attribute the high RI sensitivity of AgDNP@Au to the thinness of the Au layer (∼1 nm) and a possible sharpening of nanoparticle tips during the coating process (see TEM images in Figures 1b and S1). However, the FOM is reduced to 6.0 ± 0.5 after Au coating, as a result of a broader LSPR band. This spectral broadening may be due to a combination of plasmonic damping and slightly higher nanoparticle inhomogeneity. Indeed, compared to AgDNP, we noted a larger standard deviation of RI sensitivity values across the three synthetic batches. Increased inhomogeneity is perhaps unsurprising, given the accumulation of deviations over a two-step core–shell synthesis.

Overall, we found that both AgDNP and AgDNP@Au showed a comparable, if not superior, sensing potential among the highest-performing colloidal nanoparticle optical sensors reported to date, despite similar elemental composition (Table 1). With the enhanced chemical and colloidal stability afforded by the ultrathin Au shell, the AgDNP@Au shows promise for interfacing with, and sensing, proteins at their surface.

### Nanoparticle Surface Chemistry for Specific and Modular Protein Immobilization

The Au layer over the AgDNP core not only improves the stability of these nanoparticles in biological buffers but also permits covalent Au-thiol chemistry,^41,42^ a versatile approach to introducing surface ligands. The surface ligands that create ideal nano-bio interfaces should fulfill three conditions: (1) maintain the colloidal stability of the nanoparticle in the biological buffers used for protein–protein sensing, (2) minimize the nonspecific interaction of proteins with particle surfaces,^43^ and (3) allow for immobilization of selected proteins through specific binding. With these considerations, a known strategy for engineering nanoparticle surfaces for nano-bio interactions involves a combination of “stabilizing ligands” and “linker ligands” to achieve high colloidal stability, modular design, and specific protein immobilization.

Thiolated polyethylene glycol (PEG-SH) and its derivatives are commonly used surface ligands for stabilizing noble metal nanoparticles in buffers and conferring biocompatibility.^44,45^ Attaching PEG molecules with high molecular weights (MW) typically improves the colloidal stability of nanoparticles and reduces nonspecific interactions between proteins and particle surfaces through steric hindrance.^46,47^ However, larger PEG ligands would also reduce the nanoparticle’s ability to sense biomolecules, as longer surface ligands would restrict access of analyte biomolecules to the LSPR hotspots at the nanoparticle surface.^48^ Given this trade-off, we employed methoxy PEG thiol (mPEG-SH) with low MW (∼0.8 kDa, “PEG800”) to function as the “stabilizing ligand”, while also including Tween 20 in the washing and protein–protein sensing steps to effectively reduce particle loss caused primarily by sticking to container walls.^48^

**Figure 2:**
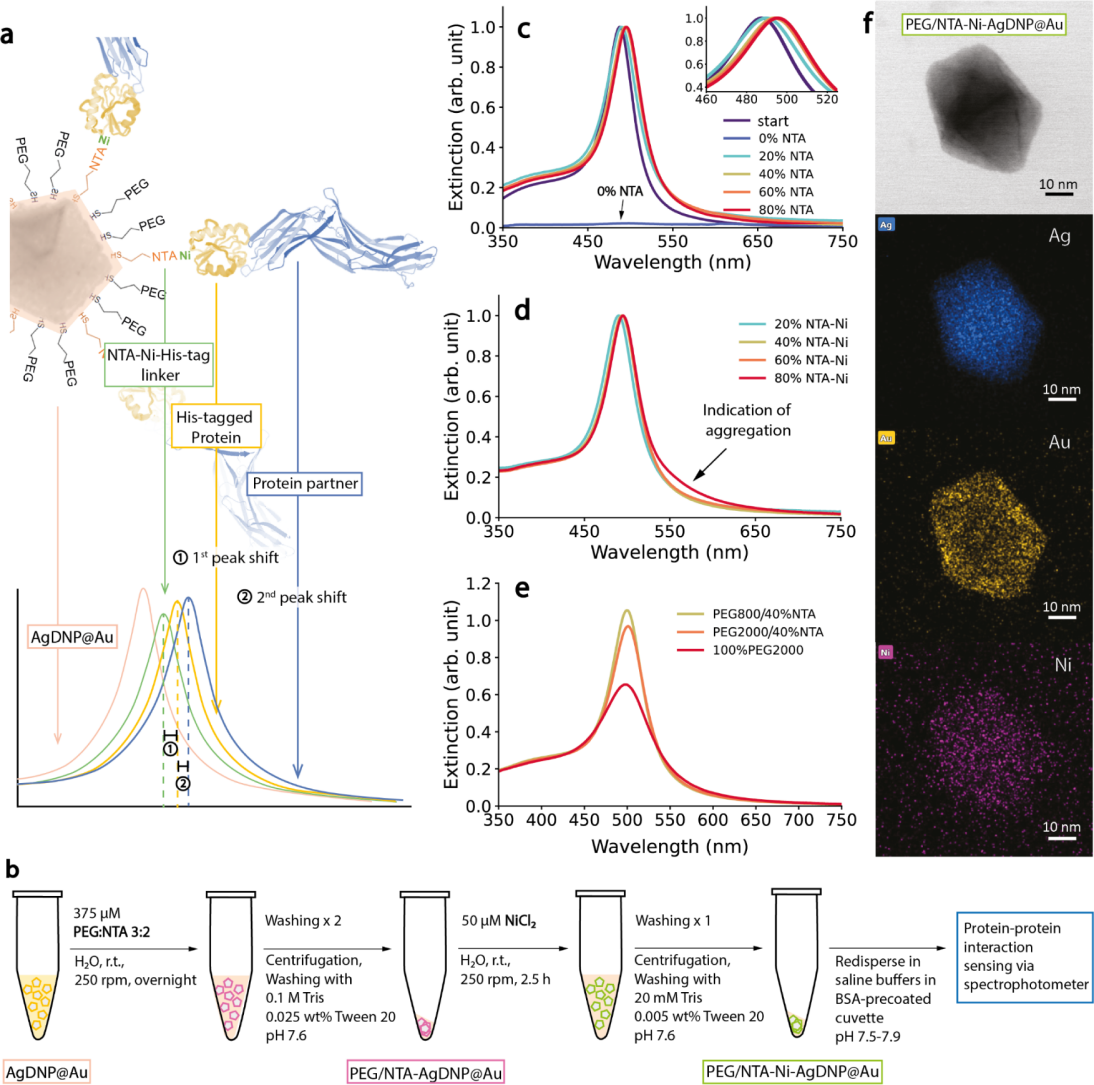
Surface modification of AgDNP@Au for protein–protein interaction sensing. (a) Schematic of protein–protein interaction sensing via NTA-Ni-His-tag linker and the stepwise change in LSPR band from refractive-index sensing. The “1^st^ peak shift” and “2^nd^ peak shift” are defined as the spectral shift after the attachment of the His-tagged protein and the protein partner, respectively. (b) Schematic of workflow of particle surface modification. Rpm refers to shaking speeds during incubation steps (see Supporting Information). (c) The effect of NTA:PEG800 ratio on LSPR peak after surface modification. The total ligand to nanoparticle ratio and total amount were kept the same. Nanoparticles were redispersed in H_2_O for the measurements. The spectra were normalized except for 0% NTA (100% PEG800)-modified particles, which could not be redispersed. Insert: zoom-in of the spectral peak. (d) Extinction spectra of nanoparticles after PEG800/(20–80%)NTA-Ni surface modification. Particles were redispersed in 20 mM HEPES, 150 mM NaCl, 0.025 wt % Tween 20, pH 7.5 buffer. (e) Effect of mPEG-SH length used in PEG/NTA surface modification on LSPR peak. For comparison, the spectrum of nanoparticles modified with 100% PEG2000 is shown. (f) STEM-EDX images of a representative PEG/NTA-Ni modified AgDNP@Au. 80% NTA-Ni was used to improve detectability of Ni. From top to bottom: bright-field image, Ag channel (blue), Au channel (yellow) and Ni channel (pink).

These “stabilizing ligands” can then be used in combination with “linker ligands” to specifically tether proteins to the nanoparticle surface. Several different strategies are applied in both chip-based SPR and nanoparticle-based LSPR sensing technologies to bind biomolecules to gold surfaces. Common linkers include biotin–streptavidin linkage,^48^ protein-A/G-IgG linkage,^49^ and Ni(II)-nitrilotriacetic acid (NTA)-poly(6)-Histidine (“NTA-Ni-His-tag”) linkage.^50−52^ In this work, we chose to immobilize Ni(II)NTA functional groups onto the AgDNP@Au surface in order to bind proteins as close as possible to the nanosensor for maximal spatial overlap with the LSPR hotspot (Figure 2a). Specifically, we used a thiolated alkane-PEG-NTA chelated with Ni(II) (“NTA-Ni”) to bind to the His-tag of recombinantly produced proteins.^53^ With a low molecular weight of only ∼1.5 kDa including a His_6_-tag, this linkage is significantly smaller than those of other common linkers such as biotin–streptavidin (∼53 kDa), bringing the protein analyte much closer to the nanoparticle surface. Moreover, NTA-Ni-His-tag linkage allows for straightforward modular applicability to many recombinant proteins, which are routinely purified using immobilized metal ion affinity chromatography, based on the same principle.^54^

A schematic of the workflow to produce surface-modified Au-coated AgDNPs for protein–protein interaction sensing is given in Figure 2b. With two incubation and washing steps, the AgDNP@Au surface was prepared with both mPEG-SH (“stabilizing”) and NTA-Ni (“linker”) ligands. In order to select the optimal NTA:PEG800 ratio for high colloidal stability and sufficiently high specific linkage sites, the NTA content was varied (20–80%) while keeping the total applied ligands in constant excess (375 μM ligands to ∼0.1 nM NPs; for estimation, see Text S1, eqs S3–S5 and Text S2, eq S6). As shown in Figure 2c, nanoparticles modified with only PEG800 could not be redispersed in water. With only 20% NTA, however, nanoparticle colloidal stability was significantly enhanced, and a +2.0 nm (−10.4 meV) redshift along with a peak width broadening by 23%, were observed. Higher NTA ratios of 40–80% yielded similar but more significant redshifts of +6.6 to −8.0 nm (−34.0 to −41.1 meV), which reflected successful surface ligand attachment providing adequate specific linkage sites. In addition, compared to 20% NTA, peaks experienced less broadening (12.5–15.3%), suggesting reduced plasmonic damping with increasing NTA and decreasing PEG800. Finally, electron energy loss spectroscopy (EELS) confirmed the appearance of sulfur and an intensified nitrogen signal on the modified particle surface after ligand incorporation (Figure S6).

We further investigated PEG800/NTA-Ni surface-modified AgDNP@Au after incubation in excess NiCl_2_ (50 μM NiCl_2_ to ∼0.1 nM NPs, also see Text S1, eqs S3–S5 and Text S2, eq S6). In Figure 2d, particles modified with 20–60% NTA-Ni (i.e., 80–40% PEG800 correspondingly) were well redispersible in HEPES saline while maintaining their spectra. Dynamic light scattering (DLS) did not indicate any aggregate formation (Figure S7) after PEG800/40%NTA-Ni modification, and also revealed a nanoparticle hydrodynamic diameter increase of ∼3.1 nm, similar to previous findings for Au nanospheres functionalized with similar ligands.^51,55^ With 80% NTA-Ni, however, a shoulder appeared in the spectrum at ∼550 nm upon nanoparticle redispersion in HEPES saline, indicating the formation of nanoparticle aggregates.^56^ Taken together, the spectra of Figure 2c,d and DLS measurements indicate that 40–60% NTA, with corresponding 60–40% PEG800, provided adequate linker sites, while also maintaining colloidal stability of AgDNP@Au in saline buffers after Ni(II)-NTA conjugation. 40% NTA-Ni was selected as a promising ligand combination for further application as particles were not susceptible to aggregation or LSPR peak broadening, even when larger mPEG-SH (MW ∼ 2 kDa, “PEG2000”) was used as the stabilizing ligand, which tends to lead to broadened spectra that lower optical sensing performance (Figure 2e).

To validate the successful conjugation of Ni(II) to surface NTA ligands, we characterized the elemental composition of the surface-modified nanoparticles with scanning transmission electron microscopy (STEM)-EDX. Figures 2f and S8 show that the Ni signal was detected on nanoparticle surfaces after incubation in NiCl_2_ solution and redispersion in a HEPES buffer. The zeta potential shifted slightly from −32.7 ± 0.9 mV for PEG800/40%NTA-Ni-modified nanoparticles to −28.3 ± 1.2 mV, which aligns with Ni precharging of surface NTA.^53^ Given these observations, we utilized the PEG800/40%NTA-Ni surface-modified AgDNP@Au (subsequently referred to as “AgDNP@Au nanobiosensors”) for protein–protein interaction sensing measurements.

### Measuring Protein–protein Interactions with Nanobiosensors in Solution

We demonstrate the capabilities of AgDNP@Au nanobiosensors using two sets of well-studied protein interaction pairs. The first is the interaction between the intrinsically disordered transcriptional activation domain of the tumor suppressor protein p53 (“TAD”) and the molten-globular nuclear receptor coactivator binding domain of the CREB-binding protein (“NCBD”).^57,58^ The second protein pair is the interaction between the intrinsically disordered N-terminal domain of the cytosolic peroxisomal targeting protein PEX5 (“PEX5”) and the globular N-terminal domain of the peroxisomal membrane-associated import protein PEX14 (“PEX14”).^59−61^ Specifically, we measured two PEX5 constructs, PEX5 (1–113) and PEX5 (110–230), and one PEX14 (16–80) construct. All protein constructs were in the molar mass range of 7–15 kDa (see Table S1).

As depicted in Figure 2a, protein–protein interactions were measured in the solution phase. First, the nanobiosensors were redispersed in biological buffers, where their unperturbed spectra suggested good colloidal stability. Next, the His-tagged protein (His_6_-tag on the N-terminus of TAD and PEX5, “hTAD” and “hPEX5”) was added to the nanobiosensors to bind to the NTA-Ni via NTA-Ni-His-tag coordination chemistry. This initial conjugation was allowed to approach equilibrium as tracked by the spectral shift of the LSPR extinction spectrum (“first peak shift”, typically achieved within 20 min). After this, the partner protein was manually added, and the spectrum was monitored kinetically, and/or near equilibrium, to extract binding information (“second peak shift”). We note that intermediate purification steps were not needed, underscoring this technique’s straightforward nature and ease of use.

### Measuring Specific Protein–Protein Interactions

**Figure 3:**
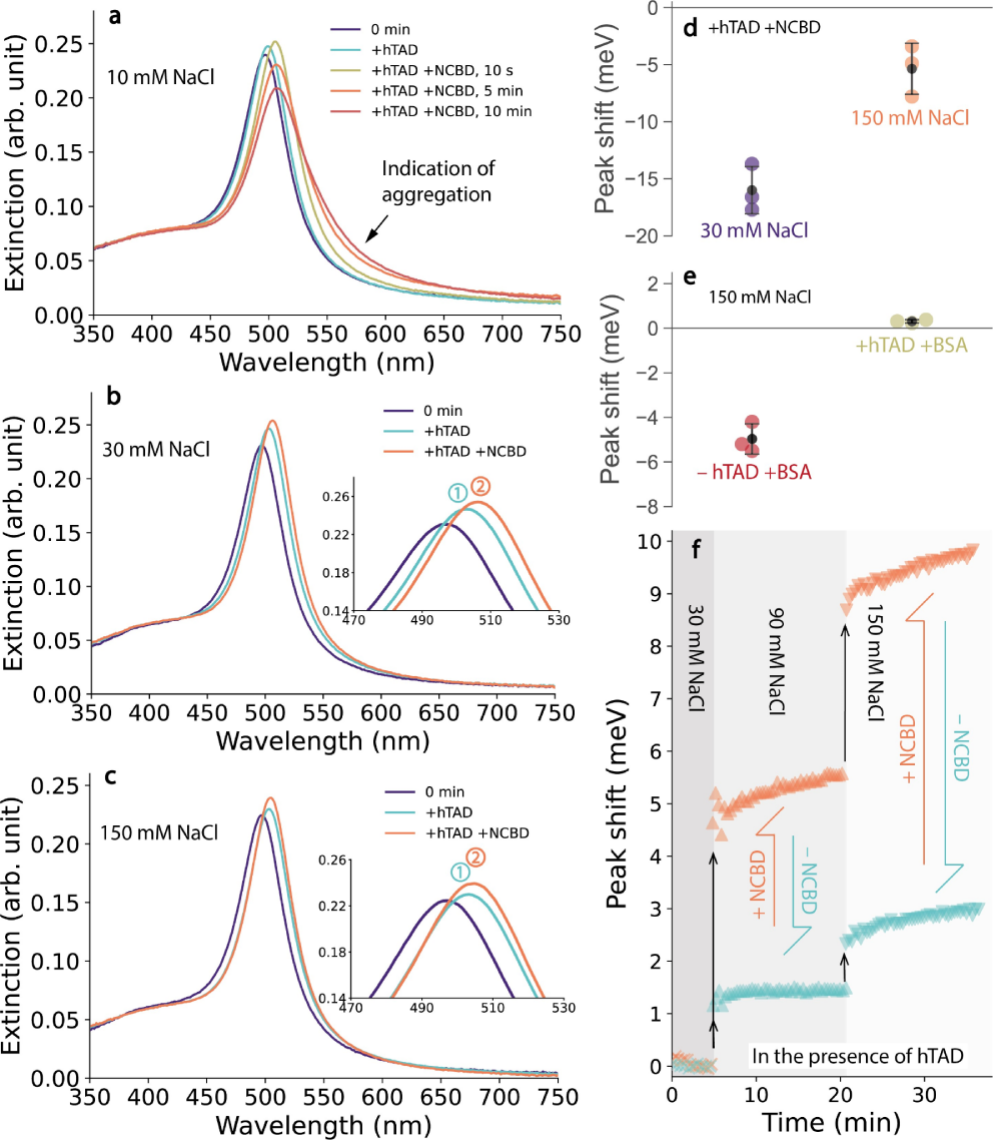
Measuring His-Tagged TAD (“hTAD”, 0.16 μM) and NCBD (0.27 μM) interaction in 10 mM Tris buffer, 0.025 wt % Tween 20, pH 7.5, containing (a) 10 mM NaCl, (b) 30 mM NaCl and (c) 150 mM NaCl. The nanobiosensor concentration was kept at an estimated ∼0.025 nM. Inset of (b) and (c): zoom-in of spectral peaks. Spectral intensities were corrected for the dilution effect from protein addition. ① and ② indicate the 1st and 2nd peak shifts, respectively. (d) The 2nd peak shifts after addition of NCBD to hTAD-immobilized nanobiosensors at 25 min (near equilibrium) in Tris buffer containing 30 and 150 mM NaCl. (e) The peak shifts after addition of 1.6 μM BSA to hTAD-immobilized and bare nanobiosensors at 25 min (near equilibrium) in Tris buffer containing 150 mM NaCl. Black dots and error bars in (d) and (e) represent the mean values and standard deviations of triplicate experiments, respectively. (f) Stepwise LSPR-peak blue shifts after changing NaCl concentration from 30 mM to 90 mM, then to 150 mM. The salt concentration effect was measured for nanobiosensors attached either with hTAD alone (“–NCBD”) or with hTAD and NCBD (“+NCBD”), for comparison.

In Figure 3a–c, we validate the sensing of the interaction between His-tagged TAD (“hTAD”, 7.0 kDa) and NCBD (6.9 kDa) by recording the spectral changes of the nanobiosensors upon the two-step protein addition in Tris-based buffers at different NaCl concentrations. At 10 mM NaCl (Figure 3a), we observed a large redshift of +6.2 nm (−30.4 meV) after 10 s of manual mixing, but several minutes afterward, the peak broadened with a red shoulder and reduced intensity, an indication of nanoparticle aggregation.^56^ This behavior can be explained as follows. Kim et al. reported the highest binding affinity of TAD and NCBD at 10 mM NaCl (*K*_D_ = 0.104 μM) among the three NaCl concentrations tested here (10 mM, 30 mM, and 150 mM).^58^ Under these conditions, multiple contacts could occur between a single soluble NCBD protein and several TAD-nanoparticle complexes,^58^ leading to the formation of nanoparticle oligomers.

When increasing the NaCl concentration to 30 and 150 mM, the second peak red-shift decreased to +3.2 nm (−15.5 meV) and +1.0 nm (−4.9 meV) (Figure 3b–d). These findings match the reported trend of reduced binding affinities at higher salt concentrations, as a larger red-shift is expected for lower equilibrium dissociation constant *K*_D_ as more NCBD binds to nanoparticle-immobilized hTAD at the same solution NCBD concentration (*K*_D_ = 1.6 μM at 30 mM NaCl to 29.9 μM at 150 mM NaCl).^58^ The spectral line shapes remained narrow throughout the measurements, indicating stable colloidal dispersions.

To further demonstrate that these spectral shifts arose from the protein–protein interactions themselves rather than NaCl, we monitored the spectrum of a single nanobiosensor sample containing hTAD and NCBD undergoing the addition of NaCl (Figure 3f). As expected, we observed rapid, stepwise blue shifts in the LSPR peak position when increasing the NaCl concentration first from 30 mM to 90 mM, then to 150 mM, indicative of enhanced dissociation at higher salt concentrations. Much smaller blue shifts were observed for nanobiosensors immobilized with hTAD alone in the absence of NCBD (Figure 3f). Altogether, these results indicate that the specific protein interaction between hTAD and NCBD is primarily responsible for the observed LSPR spectral changes in Figure 3.

As a final verification of the specificity of the protein–protein interaction detected, we performed control experiments where the protein binding partner (NCBD) was replaced by a protein not expected to bind to TAD: bovine serum albumin (BSA, 66.5 kDa). As shown in Figure 3e, the addition of BSA to hTAD-immobilized nanoparticles led to essentially no spectral shift, confirming the lack of binding between BSA and TAD, in complete contrast to the addition of NCBD to TAD (Figure 3d). We also show that BSA addition to AgDNP@Au nanobiosensors in the absence of hTAD resulted in a gradual but significant redshift in the LSPR position (Figure 3e). This points to some nonspecific interactions between BSA and the modified nanoparticle surface, in agreement with previous studies showing that PEG alone, particularly at low molecular weight, generally cannot fully prevent protein adsorption to Au nanoparticle surfaces.^53,62^ However, with sufficient hTAD loading, nonspecific interaction sites on the nanoparticle surface appear to be minimized, permitting the detection of specific protein–protein interactions. We note that contributions of BSA, or any protein without a metallic or chromophore cofactor, to the extinction spectrum are negligible compared to the extinction of the nanoparticle LSPR at the concentrations used in this study.

**Figure 4:**
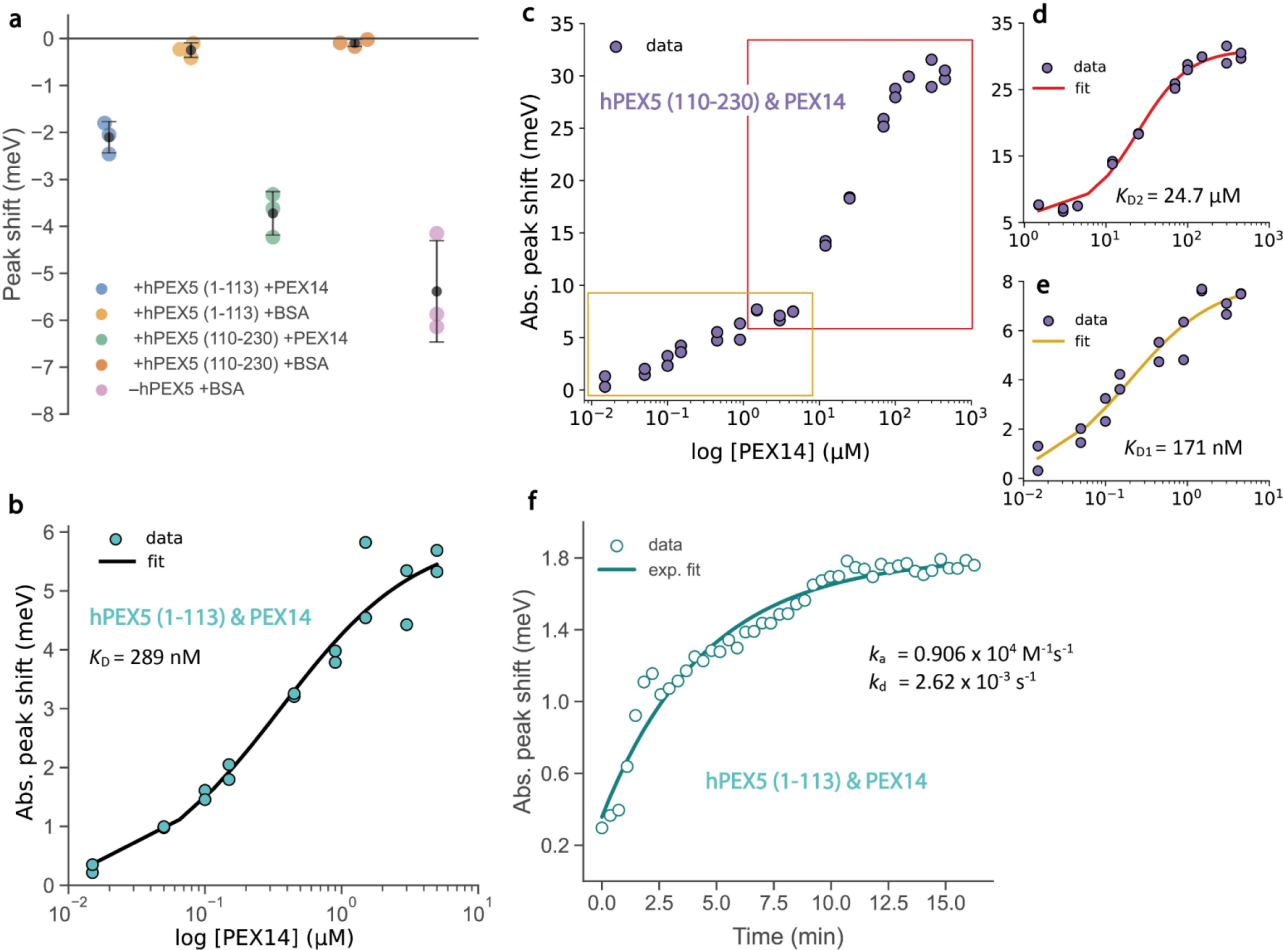
Quantifying the PEX5-PEX14 interaction. (a) LSPR peak shifts caused by addition of 0.15 μM PEX14 (blue and green dots) or 0.15 μM BSA (gold and orange dots) to nanobiosensors decorated with hPEX5 (1–113) or hPEX5 (110–230) at 0.015 μM. The peak shifts caused by 0.15 μM BSA addition to nanobiosensors without any PEX5 are shown for comparison (violet dots). Black dots and error bars represent the mean values and standard deviations of triplicate experiments, respectively. (b) The binding curve of PEX5 (1–113) and PEX14 (16–80) pair. (c) The binding curve of PEX5 (110–230) and PEX14 (16–80) pair. (d,e) Zoomed-in view of the high-concentration (d) and low-concentration (e) parts of the full binding curve in (c). Hill–Waud equation (eqs S1, S2) was used for the fittings in (b–e). (f) A typical kinetic measurement of the PEX5 (1–113) and PEX14 (16–80) interaction without premixing at a PEX14:PEX5 ratio of 10:1. For each measurement, 0.015 μM hPEX5 construct was added to ∼0.025 nM nanobiosensors in a 20 mM sodium phosphate buffer containing 100 mM NaCl and 0.025 wt % Tween 20 at pH 7.9. The solid line is an exponential fit through the data.

In similar experiments, we demonstrate the binding specificity between both PEX5 constructs and PEX14 using BSA (Figure 4a). Similar to hTAD, the attachment of either His-tagged PEX5 construct (“hPEX5”), hPEX5 (1–113), or hPEX5 (110–230), prevented nonspecific binding of BSA with the nanoparticle surface. However, significant peak redshifts were observed in the presence of the PEX5 binding partner PEX14 (16–80). Perhaps more substantially, these measurements demonstrate the modularity of this sensing platform, as the same nanobiosensors could be applied to either protein binding pairs, without any additional modification, to sense their specific interactions.

### Quantitative Measurement of Protein–Protein Binding Parameters

Beyond the qualitative detection of protein–protein interactions, we investigated the use of these nanobiosensors for quantifying binding affinities and kinetics. Figure 4b–e shows the magnitude of shifts in the LSPR peak position caused by the addition of different concentrations of PEX14 (16–80) to either of the two His-tagged PEX5 constructs tethered to the nanobiosensors. The nanoparticles maintained colloidal stability throughout the entire binding curves, spanning a dynamic range of up to 5 orders of magnitude in PEX14 concentration (see select spectra in Figures S9 and S10). The equilibrium dissociation constant (*K*_D_) can be derived from fits to each of the binding curves using the Hill–Waud equation (eq S1, also see Supporting Information). *K*_D_ of PEX5 (1–113) and PEX14 was determined to be 289 nM, which is close to values reported by isothermal titration calorimetry (ITC, 157 nM) and surface plasmon resonance (SPR, 87 nM) (see Table 2).^59^ It is well-known that measurements of *K*_D_ for the same protein pair can vary, sometimes by 2-fold or more, for different techniques or even the same technique under slightly different conditions.^63^ Hence, our AgDNP@Au nanobiosensors appear capable of measuring *K*_D_ in a fashion comparable with established techniques. Differences in the buffer solution, e.g., the presence of Tween 20 and a more basic pH, the presence of the His-tag on PEX5, and, of course, the presence of nanoparticles, may also have contributed to the slight discrepancies with literature values.

**Table 2:** Comparison of the PEX5-PEX14 Binding Parameters in this Study and from Refs. (59) and (61)^a^

(His-tagged) PEX5	PEX14	This work	Reported values
1–113	16–80	*K*_D_ = 289 nM, *k*_a_ = 0.906 × 10^4^ M^–1^ s^–1^, *k*_d_ = 2.62 × 10^–3^ s^–1^	*K*_D_ (ITC) = 157 nM, *K*_D_ (SPR) = 87 nM, *k*_a_ = 7.0 × 10^4^ M^–1^ s^–1^, *k*_d_ = 6.1 × 10^–3^ s^–1^
110–230	16–80	*K*_D1_ = 171 nM *K*_D2_ = 24.7 μM	*K*_D1_ (ITC) = 139–344 nM *K*_D2_ (ITC) = 6.31 μM

a Note 1: In Ref. (59), PEX5 (1–117) was used instead of PEX (1–113). However, the same binding motifs were involved. Note 2: The binding between PEX5 (1–113) and PEX14 only concerns the interaction between the W0 motif and PEX14, whereas multiple motifs of PEX5 (110–230) can interact with PEX14. *K*_D1_ of the initial binding stage seems to reflect the sum of the interaction between PEX5 W1–W3 motif and PEX14, whereas *K*_D2_ seems to reflect the binding between the W4 motif and PEX14 (see Ref. (59) for more details). Note 3: These references are co-authored by one or more of the co-authors of this study.

The kinetic change in the LSPR peak for PEX5 (1–113) and PEX14 binding is shown in Figure 4f. The average observable rate *k*_obs_ was 0.00398 ± 0.00220 s^–1^, as determined from the average of three separate measurements and their fits to exponential functions (see also Figure S13). The association and dissociation rate constants *k*_a_ and *k*_d_ were derived from *k*_obs_ and *K*_D_ to be 0.906 × 10^4^ M^–1^ s^–1^ and 2.62 × 10^–3^ s^–1^, respectively. These values are 7.7-fold and 2.3-fold smaller than the values reported by SPR (see Table 2).^59^ While the measured dissociation rate *k*_d_ is close to the literature value, the association rate *k*_a_ is smaller, most likely due to diffusion limitations from inadequate manual mixing.

As described in Gopalswamy et al., PEX5 (1–113) contains a single motif W0, whereas PEX5 (110–230) carries four diaromatic peptide motifs W1–W4 that can interact with PEX14 (16–80).^61^ Motifs W1–W3 bind strongly to PEX14, showing small *K*_D_ values in a narrow range of 139–344 nM, as measured by ITC. The farthest motif, W4, interacts much more weakly with PEX14 and exhibits a *K*_D_ of 6.3 μM. These disparate *K*_D_ values appear to be reflected in the two inflection points of the binding curve of PEX5 (110–230) and PEX14 in Figure 4c.

Each inflection point can be analyzed separately using the Hill–Waud equation (eq S1) to give *K*_D1_ = 171 nM and *K*_D2_ = 24.7 μM (Figure 4d,e). *K*_D1_ can be attributed to the strong binding between motifs W1–W3 of PEX5 (110–230) and PEX14. *K*_D2_ seems to be slightly underestimated compared to the literature,^61^ which can be rationalized by the larger distance of W4 to the nanoparticle surface compared to other motifs (the His_6_-tag is at the N-terminus of the PEX5 construct and therefore closer to the W1 motif). Given that the LSPR sensitivity is highest at the nanoparticle surface and decays exponentially away from the surface, sensing could be diminished at farther motifs. We also cannot rule out the contribution of nonspecific interactions with the nanoparticle at such high protein concentrations and, of course, perturbations arising from protein immobilization to a nanoparticle. Finally, we note that evidently different Hill’s coefficients were observed for the first and second inflection points (*n*_1_ = 0.838 < 1, *n*_2_ = 1.19 > 1) of PEX5 (110–230) and PEX14 (Figure S12, eqs S1,S2). This difference may not be surprising as the first inflection point corresponds to three binding sites and the second to one. However, pinpointing the underlying mechanism would require more detailed structural studies, which are beyond the scope of this study.

As with the PEX5 (1–113) and PEX14 pair, the binding kinetics of the PEX5 (110–230) and PEX14 could also be measured using the AgDNP@Au nanobiosensors. Near the first inflection point in the binding curves of PEX5 (110–230) and PEX14, we find *k*_a_ = 1.24 × 10^4^ M^–1^ s^–1^ and *k*_d_ = 2.12 × 10^–3^ s^–1^ (Figure S11). These rates are similar to that of PEX5 (1–113) and PEX14, though with the same potential mixing caveat. Overall, the AgDNP@Au nanobiosensors have proven to be capable of sensing various protein–protein interactions in the solution phase both qualitatively and quantitatively, and both near equilibrium and kinetically.

## Conclusions

In this study, we have presented a simple and elegant optical sensing technique for measuring protein–protein interactions using colloidal nanoparticles in a liquid suspension. This technique is based on Ag decahedral nanoparticles coated with a thin layer of Au, which are chemically and colloidally stable in biological buffers, possess excellent optical sensitivity and sensing figures-of-merit, and permit straightforward surface functionalization chemistry. In order to target specific protein–protein interactions, we have used a combination of surface ligands (PEG800, NTA) that serve to minimize the distance of proteins to the nanoparticle surface, reduce nonspecific binding, and specifically bind to polyhistidine tags of recombinant proteins. With these nanobiosensors, we were able to detect the binding between two sets of small globular proteins, TAD–NCBD and PEX5–PEX14, demonstrating specificity, straightforward modularity, and quantification of binding parameters in good agreement with literature values.

This work greatly extends prior efforts to utilize the highly sensitive LSPR of metallic nanoparticles for biomolecular sensing in the solution phase. Metallic nanoparticles adherent to surfaces are, unfortunately, susceptible to artifacts related to the nearby extended surface.^23^ Prior efforts to use nanoparticles in liquid suspension have been limited to measuring lipid–protein interactions.^24,64^ In this study, we have shown that our design, which integrates several advancements in nanomaterials synthesis and surface modification, permits the measurement of specific protein–protein interactions with performance comparable to popular methods such as surface plasmon resonance (SPR) and isothermal titration calorimetry (ITC). Furthermore, this technique does not require special instrumentation or amino acid modification, demands little protein sample, and avoids extended surfaces. These features, combined with its relative cost-effectiveness, make this technique a complementary, or even preferred, alternative to other established methods.

This solution LSPR sensing platform is not without its challenges. Some challenges are inherent to many biosensing technologies, such as the optimization of buffer conditions, care taken to avoid nonspecific interactions, and taking into account the immobilization of one protein partner. Other challenges are inherent to the use of nanoparticles in the solution phase, such as issues with colloidal stability and manual mixing. The use of alternative surface ligands or ligand combinations and an automated injection setup should mitigate these challenges. An additional consideration arises from the tight LSPR confinement to the nanoparticle tips. This tight confinement contributes to the high sensitivity of the particles but also means that the sensing volume is optimized for protein pairs of particular molecular weight combinations. This molecular weight dynamic range remains to be determined.

Beyond the measurement of purified proteins in solution, the customizability of our platform suggests extension to other applications. For example, the ligand chemistry can be adapted to immobilize other types of biomolecules, such as nucleic acids, to study a variety of different biomolecular interactions. Improved surface chemistry may also permit measurements in complex biological mixtures, such as serum, cell extract, solutions under oxidizing conditions, and potentially even live cells or tissue.

## Supporting Information Available

Materials and methods in greater detail, supporting data figures, supporting table, supporting text, and calculations (PDF)

## References

[ref1] Braun P.; Gingras A.-C. History of Protein-Protein Interactions: From Egg-White to Complex Networks. Proteomics 2012, 12 (10), 1478–1498. 10.1002/pmic.201100563. 22711592

[ref2] Pardee A. B. Regulatory Molecular Biology. Cell Cycle 2006, 5 (8), 846–852. 10.4161/cc.5.8.2634. 16552190

[ref3] Kuzmanov U.; Emili A. Protein-Protein Interaction Networks: Probing Disease Mechanisms Using Model Systems. Genome Med. 2013, 5 (4), 37 10.1186/gm441. 23635424 PMC3706760

[ref4] Luck K.; Kim D.-K.; Lambourne L.; Spirohn K.; Begg B. E.; Bian W.; Brignall R.; Cafarelli T.; Campos-Laborie F. J.; Charloteaux B.; Choi D.; Coté A. G.; Daley M.; Deimling S.; Desbuleux A.; Dricot A.; Gebbia M.; Hardy M. F.; Kishore N.; Knapp J. J.; Kovács I. A.; Lemmens I.; Mee M. W.; Mellor J. C.; Pollis C.; Pons C.; Richardson A. D.; Schlabach S.; Teeking B.; Yadav A.; Babor M.; Balcha D.; Basha O.; Bowman-Colin C.; Chin S.-F.; Choi S. G.; Colabella C.; Coppin G.; D’Amata C.; De Ridder D.; De Rouck S.; Duran-Frigola M.; Ennajdaoui H.; Goebels F.; Goehring L.; Gopal A.; Haddad G.; Hatchi E.; Helmy M.; Jacob Y.; Kassa Y.; Landini S.; Li R.; van Lieshout N.; MacWilliams A.; Markey D.; Paulson J. N.; Rangarajan S.; Rasla J.; Rayhan A.; Rolland T.; San-Miguel A.; Shen Y.; Sheykhkarimli D.; Sheynkman G. M.; Simonovsky E.; Taşan M.; Tejeda A.; Tropepe V.; Twizere J.-C.; Wang Y.; Weatheritt R. J.; Weile J.; Xia Y.; Yang X.; Yeger-Lotem E.; Zhong Q.; Aloy P.; Bader G. D.; De Las Rivas J.; Gaudet S.; Hao T.; Rak J.; Tavernier J.; Hill D. E.; Vidal M.; Roth F. P.; Calderwood M. A. A Reference Map of the Human Binary Protein Interactome. Nature 2020, 580 (7803), 402–408. 10.1038/s41586-020-2188-x. 32296183 PMC7169983

[ref5] Hein M. Y.; Hubner N. C.; Poser I.; Cox J.; Nagaraj N.; Toyoda Y.; Gak I. A.; Weisswange I.; Mansfeld J.; Buchholz F.; Hyman A. A.; Mann M. A Human Interactome in Three Quantitative Dimensions Organized by Stoichiometries and Abundances. Cell 2015, 163 (3), 712–723. 10.1016/j.cell.2015.09.053. 26496610

[ref6] Zhou M.; Li Q.; Wang R. Current Experimental Methods for Characterizing Protein-Protein Interactions. ChemMedChem 2016, 11 (8), 738–756. 10.1002/cmdc.201500495. 26864455 PMC7162211

[ref7] Pierce M. M.; Raman C. S.; Nall B. T. Isothermal Titration Calorimetry of Protein–Protein Interactions. Methods 1999, 19 (2), 213–221. 10.1006/meth.1999.0852. 10527727

[ref8] Göbl C.; Madl T.; Simon B.; Sattler M. NMR Approaches for Structural Analysis of Multidomain Proteins and Complexes in Solution. Prog. Nucl. Magn. Reson. Spectrosc. 2014, 80, 26–63. 10.1016/j.pnmrs.2014.05.003. 24924266

[ref9] Sugiki T.; Kobayashi N.; Fujiwara T. Modern Technologies of Solution Nuclear Magnetic Resonance Spectroscopy for Three-Dimensional Structure Determination of Proteins Open Avenues for Life Scientists. Comput. Struct. Biotechnol. J. 2017, 15, 328–339. 10.1016/j.csbj.2017.04.001. 28487762 PMC5408130

[ref10] Heyduk T.; Ma Y.; Tang H.; Ebright R. H. Fluorescence Anisotropy: Rapid, Quantitative Assay for Protein-DNA and Protein-Protein Interaction. Methods Enzymol. 1996, 274, 492–503. 10.1016/S0076-6879(96)74039-9. 8902827

[ref11] Martin S. F.; Tatham M. H.; Hay R. T.; Samuel I. D. W. Quantitative Analysis of Multi-Protein Interactions Using FRET: Application to the SUMO Pathway. Protein Sci. 2008, 17 (4), 777–784. 10.1110/ps.073369608. 18359863 PMC2271167

[ref12] Jerabek-Willemsen M.; André T.; Wanner R.; Roth H. M.; Duhr S.; Baaske P.; Breitsprecher D. MicroScale Thermophoresis: Interaction Analysis and beyond. J. Mol. Struct. 2014, 1077, 101–113. 10.1016/j.molstruc.2014.03.009.

[ref13] Crabtree M. D.; Shammas S. L. Stopped-Flow Kinetic Techniques for Studying Binding Reactions of Intrinsically Disordered Proteins. Methods Enzymol. 2018, 611, 423–457. 10.1016/bs.mie.2018.09.026. 30471695

[ref14] Dietz M. S.; Wehrheim S. S.; Harwardt M.-L. I. E.; Niemann H. H.; Heilemann M. Competitive Binding Study Revealing the Influence of Fluorophore Labels on Biomolecular Interactions. Nano Lett. 2019, 19 (11), 8245–8249. 10.1021/acs.nanolett.9b03736. 31621335

[ref15] Bobone S.; Storti C.; Fulci C.; Damiani A.; Innamorati C.; Roversi D.; Calligari P.; Pannone L.; Martinelli S.; Tartaglia M.; Bocchinfuso G.; Formaggio F.; Peggion C.; Biondi B.; Stella L. Fluorescent Labeling Can. Significantly Perturb Measured Binding Affinity and Selectivity of Peptide-Protein Interactions. J. Phys. Chem. Lett. 2024, 15 (40), 10252–10257. 10.1021/acs.jpclett.4c01767. 39360979

[ref16] Soltermann F.; Struwe W. B.; Kukura P. Label-Free Methods for Optical in Vitro Characterization of Protein–protein Interactions. Phys. Chem. Chem. Phys. 2021, 23 (31), 16488–16500. 10.1039/D1CP01072G. 34342317 PMC8359934

[ref17] Helmerhorst E.; Chandler D. J.; Nussio M.; Mamotte C. D. Real-Time and Label-Free Bio-Sensing of Molecular Interactions by Surface Plasmon Resonance: A Laboratory Medicine Perspective. Clin. Biochem. Rev. 2012, 33 (4), 161–173. 23267248 PMC3529553

[ref18] Petersen R. L. Strategies Using Bio-Layer Interferometry Biosensor Technology for Vaccine Research and Development. Biosensors 2017, 7 (4), 49 10.3390/bios7040049. 29088096 PMC5746772

[ref19] Knezevic J.; Langer A.; Hampel P. A.; Kaiser W.; Strasser R.; Rant U. Quantitation of Affinity, Avidity, and Binding Kinetics of Protein Analytes with a Dynamically Switchable Biosurface. J. Am. Chem. Soc. 2012, 134 (37), 15225–15228. 10.1021/ja3061276. 22946661

[ref20] Schuck P.; Zhao H. The Role of Mass Transport Limitation and Surface Heterogeneity in the Biophysical Characterization of Macromolecular Binding Processes by SPR Biosensing. Methods Mol. Biol. 2010, 627, 15–54. 10.1007/978-1-60761-670-2_2. 20217612 PMC4134667

[ref21] Wang D.; Wu H.; Schwartz D. K. Three-Dimensional Tracking of Interfacial Hopping Diffusion. Phys. Rev. Lett. 2017, 119 (26), 268001 10.1103/PhysRevLett.119.268001. 29328686

[ref22] Czajka P.; Antosiewicz J. M.; Długosz M. Effects of Hydrodynamic Interactions on the Near-Surface Diffusion of Spheroidal Molecules. ACS Omega 2019, 4 (16), 17016–17030. 10.1021/acsomega.9b02618. 31646249 PMC6796493

[ref23] Wulf V.; Knoch F.; Speck T.; Sönnichsen C. Gold Nanorods as Plasmonic Sensors for Particle Diffusion. J. Phys. Chem. Lett. 2016, 7 (23), 4951–4955. 10.1021/acs.jpclett.6b02165. 27934054

[ref24] Wu H.-J.; Henzie J.; Lin W.-C.; Rhodes C.; Li Z.; Sartorel E.; Thorner J.; Yang P.; Groves J. T. Membrane-Protein Binding Measured with Solution-Phase Plasmonic Nanocube Sensors. Nat. Methods 2012, 9 (12), 1189–1191. 10.1038/nmeth.2211. 23085614 PMC3703907

[ref25] Zijlstra P.; Paulo P. M. R.; Orrit M. Optical Detection of Single Non-Absorbing Molecules Using the Surface Plasmon Resonance of a Gold Nanorod. Nat. Nanotechnol. 2012, 7 (6), 379–382. 10.1038/nnano.2012.51. 22504707

[ref26] Lee Y. H.; Chen H.; Xu Q.-H.; Wang J. Refractive Index Sensitivities of Noble Metal Nanocrystals: The Effects of Multipolar Plasmon Resonances and the Metal Type. J. Phys. Chem. C 2011, 115 (16), 7997–8004. 10.1021/jp202574r.

[ref27] Jakab A.; Rosman C.; Khalavka Y.; Becker J.; Trügler A.; Hohenester U.; Sönnichsen C. Highly Sensitive Plasmonic Silver Nanorods. ACS Nano 2011, 5 (9), 6880–6885. 10.1021/nn200877b. 21851108

[ref28] Sugawa K.; Tahara H.; Yamashita A.; Otsuki J.; Sagara T.; Harumoto T.; Yanagida S. Refractive Index Susceptibility of the Plasmonic Palladium Nanoparticle: Potential as the Third Plasmonic Sensing Material. ACS Nano 2015, 9 (2), 1895–1904. 10.1021/nn506800a. 25629586

[ref29] McPeak K. M.; Jayanti S. V.; Kress S. J. P.; Meyer S.; Iotti S.; Rossinelli A.; Norris D. J. Plasmonic Films Can Easily Be Better: Rules and Recipes. ACS Photonics 2015, 2 (3), 326–333. 10.1021/ph5004237. 25950012 PMC4416469

[ref30] Pietrobon B.; Kitaev V. Photochemical Synthesis of Monodisperse Size-Controlled Silver Decahedral Nanoparticles and Their Remarkable Optical Properties. Chem. Mater. 2008, 20 (16), 5186–5190. 10.1021/cm800926u.

[ref31] Murshid N.; Keogh D.; Kitaev V. Optimized Synthetic Protocols for Preparation of Versatile Plasmonic Platform Based on Silver Nanoparticles with Pentagonal Symmetries. Part. Part. Syst. Charact. 2014, 31 (2), 178–189. 10.1002/ppsc.201300225.

[ref32] Murshid N.; Gourevich I.; Coombs N.; Kitaev V. Gold Plating of Silver Nanoparticles for Superior Stability and Preserved Plasmonic and Sensing Properties. Chem. Commun. 2013, 49 (97), 11355–11357. 10.1039/c3cc46075d. 24129495

[ref33] Zhang L.; Zhang Y.; Ahn J.; Wang X.; Qin D. Defect-Assisted Deposition of Au on Ag for the Fabrication of Core–Shell Nanocubes with Outstanding Chemical and Thermal Stability. Chem. Mater. 2019, 31 (3), 1057–1065. 10.1021/acs.chemmater.8b04723.

[ref34] Borah R.; Verbruggen S. W. Silver–Gold Bimetallic Alloy versus Core–Shell Nanoparticles: Implications for Plasmonic Enhancement and Photothermal Applications. J. Phys. Chem. C 2020, 124 (22), 12081–12094. 10.1021/acs.jpcc.0c02630.

[ref35] Yang Y.; Liu J.; Fu Z.-W.; Qin D. Galvanic Replacement-Free Deposition of Au on Ag for Core–Shell Nanocubes with Enhanced Chemical Stability and SERS Activity. J. Am. Chem. Soc. 2014, 136 (23), 8153–8156. 10.1021/ja502472x. 24863686

[ref36] Sherry L. J.; Chang S.-H.; Schatz G. C.; Van Duyne R. P.; Wiley B. J.; Xia Y. Localized Surface Plasmon Resonance Spectroscopy of Single Silver Nanocubes. Nano Lett. 2005, 5 (10), 2034–2038. 10.1021/nl0515753. 16218733

[ref37] Celiksoy S.; Ye W.; Wandner K.; Kaefer K.; Sönnichsen C. Intensity-Based Single Particle Plasmon Sensing. Nano Lett. 2021, 21 (5), 2053–2058. 10.1021/acs.nanolett.0c04702. 33617258

[ref38] Xue B.; Wang D.; Zuo J.; Kong X.; Zhang Y.; Liu X.; Tu L.; Chang Y.; Li C.; Wu F.; Zeng Q.; Zhao H.; Zhao H.; Zhang H. Towards High Quality Triangular Silver Nanoprisms: Improved Synthesis, Six-Tip Based Hot Spots and Ultra-High Local Surface Plasmon Resonance Sensitivity. Nanoscale 2015, 7 (17), 8048–8057. 10.1039/C4NR06901C. 25869897

[ref39] Chen H.; Kou X.; Yang Z.; Ni W.; Wang J. Shape- and Size-Dependent Refractive Index Sensitivity of Gold Nanoparticles. Langmuir 2008, 24 (10), 5233–5237. 10.1021/la800305j. 18435552

[ref40] Martinsson E.; Otte M. A.; Shahjamali M. M.; Sepulveda B.; Aili D. Substrate Effect on the Refractive Index Sensitivity of Silver Nanoparticles. J. Phys. Chem. C 2014, 118 (42), 24680–24687. 10.1021/jp5084086.

[ref41] Heuer-Jungemann A.; Feliu N.; Bakaimi I.; Hamaly M.; Alkilany A.; Chakraborty I.; Masood A.; Casula M. F.; Kostopoulou A.; Oh E.; Susumu K.; Stewart M. H.; Medintz I. L.; Stratakis E.; Parak W. J.; Kanaras A. G. The Role of Ligands in the Chemical Synthesis and Applications of Inorganic Nanoparticles. Chem. Rev. 2019, 119 (8), 4819–4880. 10.1021/acs.chemrev.8b00733. 30920815

[ref42] Häkkinen H. The Gold-Sulfur Interface at the Nanoscale. Nat. Chem. 2012, 4 (6), 443–455. 10.1038/nchem.1352. 22614378

[ref43] Frutiger A.; Tanno A.; Hwu S.; Tiefenauer R. F.; Vörös J.; Nakatsuka N. Nonspecific Binding—Fundamental Concepts and Consequences for Biosensing Applications. Chem. Rev. 2021, 121 (13), 8095–8160. 10.1021/acs.chemrev.1c00044. 34105942

[ref44] Boisselier E.; Astruc D. Gold Nanoparticles in Nanomedicine: Preparations, Imaging, Diagnostics, Therapies and Toxicity. Chem. Soc. Rev. 2009, 38 (6), 1759–1782. 10.1039/b806051g. 19587967

[ref45] Dreaden E. C.; Alkilany A. M.; Huang X.; Murphy C. J.; El-Sayed M. A. The Golden Age: Gold Nanoparticles for Biomedicine. Chem. Soc. Rev. 2012, 41 (7), 2740–2779. 10.1039/C1CS15237H. 22109657 PMC5876014

[ref46] Suk J. S.; Xu Q.; Kim N.; Hanes J.; Ensign L. M. PEGylation as a Strategy for Improving Nanoparticle-Based Drug and Gene Delivery. Adv. Drug Delivery Rev. 2016, 99 (Pt A), 28–51. 10.1016/j.addr.2015.09.012. PMC4798869 26456916

[ref47] Jokerst J. V.; Lobovkina T.; Zare R. N.; Gambhir S. S. Nanoparticle PEGylation for Imaging and Therapy. Nanomedicine 2011, 6 (4), 715–728. 10.2217/nnm.11.19. 21718180 PMC3217316

[ref48] Wang Y.; van Asdonk K.; Zijlstra P. A Robust and General Approach to Quantitatively Conjugate Enzymes to Plasmonic Nanoparticles. Langmuir 2019, 35 (41), 13356–13363. 10.1021/acs.langmuir.9b01879. 31545896 PMC6798157

[ref49] Liu S.; Haller E.; Horak J.; Brandstetter M.; Heuser T.; Lämmerhofer M. Protein A- and Protein G-Gold Nanoparticle Bioconjugates as Nano-Immunoaffinity Platform for Human IgG Depletion in Plasma and Antibody Extraction from Cell Culture Supernatant. Talanta 2019, 194, 664–672. 10.1016/j.talanta.2018.10.079. 30609588

[ref50] Zhu L.; Chang Y.; Li Y.; Qiao M.; Liu L. Biosensors Based on the Binding Events of Nitrilotriacetic Acid–Metal Complexes. Biosensors 2023, 13 (5), 507 10.3390/bios13050507. 37232868 PMC10216662

[ref51] Swartz J. D.; Gulka C. P.; Haselton F. R.; Wright D. W. Development of a Histidine-Targeted Spectrophotometric Sensor Using Ni(II)NTA-Functionalized Au and Ag Nanoparticles. Langmuir 2011, 27 (24), 15330–15339. 10.1021/la202937j. 22026818

[ref52] Ahijado-Guzmán R.; Prasad J.; Rosman C.; Henkel A.; Tome L.; Schneider D.; Rivas G.; Sönnichsen C. Plasmonic Nanosensors for Simultaneous Quantification of Multiple Protein-Protein Binding Affinities. Nano Lett. 2014, 14 (10), 5528–5532. 10.1021/nl501865p. 25153997

[ref53] De M.; Rana S.; Rotello V. M. Nickel-Ion-Mediated Control of the Stoichiometry of His-Tagged Protein/nanoparticle Interactions. Macromol. Biosci. 2009, 9 (2), 174–178. 10.1002/mabi.200800289. 19127602

[ref54] Bornhorst J. A.; Falke J. J. Purification of Proteins Using Polyhistidine Affinity Tags. Methods Enzymol. 2000, 326, 245–254. 10.1016/S0076-6879(00)26058-8. 11036646 PMC2909483

[ref55] Alsadig A.; Vondracek H.; Pengo P.; Pasquato L.; Posocco P.; Parisse P.; Casalis L. L.-F. Rapid and Facile Gold-Nanoparticles-Based Assay as a Potential Spectroscopic Tool for Trastuzumab Quantification. Nanomaterials 2021, 11 (12), 3181 10.3390/nano11123181. 34947531 PMC8708960

[ref56] Doyen M.; Goole J.; Bartik K.; Bruylants G. Amino Acid Induced Fractal Aggregation of Gold Nanoparticles: Why and How. J. Colloid Interface Sci. 2016, 464, 160–166. 10.1016/j.jcis.2015.11.017. 26613335

[ref57] Lee C. W.; Martinez-Yamout M. A.; Dyson H. J.; Wright P. E. Structure of the p53 Transactivation Domain in Complex with the Nuclear Receptor Coactivator Binding Domain of CREB Binding Protein. Biochemistry 2010, 49 (46), 9964–9971. 10.1021/bi1012996. 20961098 PMC2982890

[ref58] Kim J.-Y.; Meng F.; Yoo J.; Chung H. S. Diffusion-Limited Association of Disordered Protein by Non-Native Electrostatic Interactions. Nat. Commun. 2018, 9 (1), 4707 10.1038/s41467-018-06866-y. 30413699 PMC6226484

[ref59] Neuhaus A.; Kooshapur H.; Wolf J.; Meyer N. H.; Madl T.; Saidowsky J.; Hambruch E.; Lazam A.; Jung M.; Sattler M.; et al. A Novel Pex14 Protein-Interacting Site of Human Pex5 Is Critical for Matrix Protein Import into Peroxisomes. J. Biol. Chem. 2014, 289 (1), 437–448. 10.1074/jbc.M113.499707. 24235149 PMC3879566

[ref60] Gaussmann S.; Gopalswamy M.; Eberhardt M.; Reuter M.; Zou P.; Schliebs W.; Erdmann R.; Sattler M. Membrane Interactions of the Peroxisomal Proteins PEX5 and PEX14. Front. Cell Dev. Biol. 2021, 9, 651449 10.3389/fcell.2021.651449. 33937250 PMC8086558

[ref61] Gopalswamy M.; Zheng C.; Gaussmann S.; Kooshapur H.; Hambruch E.; Schliebs W.; Erdmann R.; Antes I.; Sattler M. Distinct Conformational and Energetic Features Define the Specific Recognition of (di)aromatic Peptide Motifs by PEX14. Biol. Chem. 2023, 404 (2–3), 179–194. 10.1515/hsz-2022-0177. 36437542

[ref62] Toro-Mendoza J.; Maio L.; Gallego M.; Otto F.; Schulz F.; Parak W. J.; Sanchez-Cano C.; Coluzza I. Bioinspired Polyethylene Glycol Coatings for Reduced Nanoparticle–Protein Interactions. ACS Nano 2023, 17 (2), 955–965. 10.1021/acsnano.2c05682. 36602983

[ref63] Jarmoskaite I.; AlSadhan I.; Vaidyanathan P. P.; Herschlag D. How to Measure and Evaluate Binding Affinities. eLife 2020, 9, e57264 10.7554/eLife.57264. 32758356 PMC7452723

[ref64] Worstell N. C.; Krishnan P.; Weatherston J. D.; Wu H.-J. Binding Cooperativity Matters: A GM1-Like Ganglioside-Cholera Toxin B Subunit Binding Study Using a Nanocube-Based Lipid Bilayer Array. PLoS One 2016, 11 (4), e0153265 10.1371/journal.pone.0153265. 27070150 PMC4829222

