# Supplementary material for: Modular Nanoparticle Platform for Solution-Phase Optical Sensing of Protein–Protein Interactions
Source: ACS Appl Opt Mater. 2025 Mar 15;3(3):676–88. doi: 10.1021/acsaom.4c00486 (PMC11959585; doi:10.1021/acsaom.4c00486)
Supplement: Supplementary file 1 — ot4c00486_si_001.pdf [file ot4c00486_si_001.pdf]

Supporting Information

Modular Nanoparticle Platform for Solution-Phase Optical Sensing of Protein-Protein Interactions

*Jieying Zhou,[1] Korneel Ridderbeek,[1] Peijian Zou,[2,3] Aaron B. Naden,[4] Stefan Gaussmann,[2,3] Fangyuan Song,[1] Pascal Falter-Braun,[5,6] Euan R. Kay,[4] Michael Sattler,[2,3] Jian Cui[1,7]**

[1]Helmholtz Pioneer Campus, Helmholtz Munich, Neuherberg, Germany

[2]Institute of Structural Biology, Molecular Targets and Therapeutics Center, Helmholtz Munich, Neuherberg, Germany

[3]Bavarian NMR Center, Department of Bioscience, School of Natural Sciences, Technical University of Munich, Garching, Germany

[4]EaStCHEM School of Chemistry, University of St. Andrews, St. Andrews, U.K.

[5]Institute of Network Biology (INET), Molecular Targets and Therapeutics Center (MTTC), Helmholtz Munich, German Research Center for Environmental Health, Neuherberg, Germany.

[6]Microbe-Host Interactions, Faculty of Biology, Ludwig-Maximilians-Universität (LMU) München, Planegg-Martinsried, Germany.

[7]Department of Bioscience, School of Natural Sciences, Technical University of Munich, Garching, Germany

***Corresponding Author: jian.cui@helmholtz-munich.de**

Materials & Methods

Chemicals and materials

Unless otherwise noted, all reagents were purchased from Sigma Aldrich.

Silver nitrate (≥99.0%, 209139), hydrogen peroxide (30 wt% aq. solution, VWR Chemicals, 23615), L-arginine (≥98.0%, Thermo Scientific, A15738.22), sodium citrate tribasic dihydrate (BioUltra, 71402), and sodium borohydride (99%, 213462), polyvinylpyrrolidone (PVP, MW = 40 kDa, PVP40), gold(III) chloride trihydrate (99.9%, 520918), poly(ethylene glycol) methyl ether thiol (mPEG-SH, MW = 0.8 and 2 kDa, 729108 and 729140 respectively), 2-{2-[2-(1-mercaptoundec-11-yloxy)-ethoxy]-ethoxy}-ethoxy-nitrilotriacetic acid (HS-$(CH_2)_{11}$-EG_3-NTA TFA salt, "NTA", >95%, Prochimia Surfaces TH 007), Tween 20 (viscous liquid, cell culture tested, P2287), HEPES buffer solution (1 M, pH 7.2-7.5, Gibco 15630122), Tris-HCl buffer solution (1 M, UltraPure™ Tris-HCl buffer, pH 7.5, Gibco 15567027), phosphate buffered saline (PBS, pH 7.4, Gibco 10010023), nickel (II) chloride hexahydrate (BioReagent, N6135), sodium chloride (99.50%, S7653), bovine serum albumin (BSA, ≥99%, A0281), sodium phosphate dibasic (BioReagent, ≥99.0%, S5136), kanamycin sulfate (molecular biology grade, Serva 26899), imidazole (≥99%, I5513), isopropyl-ß-D-thiogalacto-pyranoside (IPTG, ≥99%, Carl Roth 2316.4), Tris-(2-carboxyethyl)-phosphine hydrochloride (TCEP, ≥98%, Carl Roth HN95.2), lysozyme (Serva 28263), Deoxyribonuclease I (DNase, Serva 18535), MilliQ water.

Silver decahedral nanoparticle (AgDNP) synthesis

Silver decahedral nanoparticles (AgDNP) were synthesized generally following a published protocol.[1] We used a home-built photoreactor consisting of an aluminum block with an integrated cooling system, LEDs, and vial holders. The aluminum block, containing a water inlet and outlet with a serpentine path, was connected to a 5 L water basin via a small pump at 200 L/h. 6 LEDs (455 nm ILH-ON09-DEBL-SC211-WIR200 from Intelligent LED Solutions) were screwed onto the block along with thermal paste on the LED backs. 6 vial holders were positioned on top of the LEDs. The vial holders maintained a 10 mm spacing between the LED and the bottom of the scintillation vial. The cooling ensured that solution temperatures did not increase by more than 0.1 degrees Celsius during illumination and that output light intensity was stable (140 mW at LED, 25 mW at the top of the scintillation vial). The entire reactor was placed on a shaker so that mixing could occur without the use of stirbars.

In a 20 mL glass scintillation vial, 14 mL MilliQ water was added, followed by the following compounds in the order it is listed: 0.52 mL 50 mM sodium citrate, 0.023 mL 2 mg/mL PVP-40K, 0.025 mL 5 mM L-Arginine, 0.4 mL 5 mM $AgNO_3$, 0.2 mL freshly prepared 0.1 M $NaBH_4$. After "aging" at room temperature for 50 min in the dark, a bright yellow solution was formed. The vial was then placed in the photoreactor and shaken at 250 rpm for 10 minutes with room lights off. Directly after, the 455 nm LED was turned on (140 mW at LED), and 0.3 mL 30% H_2O_2 was added while the vial was still shaking. The shaking was maintained for 30 min and then turned off. The vial was exposed to LED light for 14.5 h in total to yield the Ag decahedral nanoparticles. We have anecdotally observed that small inconsistencies in light exposure or shaking can have large effects on the final products.

Gold coating of silver decahedral nanoparticles (AgDNP@Au)

The Au coating of silver decahedral nanoparticles was based on a published protocol.[2] In brief, 3 mL 0.0128 mM $HAuCl_4$ aqueous solution was diluted from a 10 mM stock solution, and added to 3 mL AgDNP suspension at an addition rate of 0.25 mL/h over 12 h. The reaction was performed under stirring at 200 rpm at r.t. in the dark. The molar ratio of $HAuCl_4$ in the final suspension is 1/10 of the $AgNO_3$ used for preparing the silver decahedral nanoparticle suspension. The as-received core-shell nanoparticles are labeled as AgDNP@Au.

UV-Vis spectroscopy

A V-760 UV-Vis spectrophotometer (Jasco) was used for collecting the extinction spectra of nanoparticle suspensions. 1.5-mL semi-micro polystyrene cuvettes (Brand) were used for measurement.

(Scanning) transmission electron microscopy (S)TEM, electron energy loss spectroscopy (EELS), and energy-dispersive X-ray spectroscopy (EDX)

TEM images were collected with a Libra 120 (Zeiss) operating at 120 kV. Nanoparticle samples were prepared on copper grids with lacey carbon films (Agar Scientific and Electron Microscopy Sciences).

STEM measurements were performed on a Titan Themis (FEI) operated at 200 kV and equipped with a DCOR probe corrector (CEOS), a SuperX energy dispersive X-ray spectrometer (EDX), and a Gatan Enfinium electron energy loss spectrometer (EELS). High angle annular dark field (HAADF) images were acquired with a probe convergence angle of 21.2 mrad and inner/outer collection angles of 74 and 200 mrad, respectively.

EELS spectra were acquired with a collection angle of 8.1 mrad, background subtracted using a power law background, and corrected for plural scattering by Fourier ratio deconvolution.

Dynamic light scattering (DLS) and zeta potential measurements

DLS and zeta potential were measured on a Zetasizer Ultra (Malvern). Nanoparticle (and protein) samples were measured in 1 cm polystyrene cuvettes for the hydrodynamic size, and folded capillary cells (DTS1070) for the zeta potential. The multi angle dynamic light scattering (MADLS) mode was applied for the size measurements. Results were analyzed with the ZS XPLORER software.

Refractive-index (RI) sensitivity measurements

Prior to refractive-index (RI) sensitivity measurements, nanoparticles were separated from their growth solution by centrifugation at 2500 g for 15 min, and redispersed in 2 mM sodium citrate or MilliQ water. Aliquots of nanoparticle suspensions were then added to different glycerol-water mixtures, which contained 2 mM sodium citrate if used for stabilizing the nanoparticles. Reference samples without nanoparticles were used for determining the RI of

different glycerol-containing media ranging from 1.33 to 1.39, measured using a digital handheld refractometer (DR101-60, Krüss). The shift in the localized surface plasmon resonance (LSPR) peak position was plotted against the RI change. The RI sensitivities (meV/RIU) were obtained from the slopes of a linear fit to the spectral shift relative to RI change. The figures-of-merit (FOM) were calculated as the ratio of the RI sensitivities to the full widths at half maximum (FWHM) of the LSPR peak without glycerol.[3] The mean and standard deviation values reported in this study were derived from three measurements using three different synthetic batches of nanoparticles.

H_2O_2 etching experiment

Prior to the etching experiment, nanoparticles were separated from their growth solution by centrifugation at 2500 g for 30 min, and redispersed in MilliQ water. H_2O_2 was added to reach a final concentration of 1.0 M. Typically, a 6 mL freshly prepared AgDNP@Au was concentrated into a 3 mL suspension for the etching. After etching, the nanoparticles were examined by UV-Vis spectroscopy and TEM.

Testing nanoparticle stability in buffers

AgDNP or AgDNP@Au nanoparticles were first separated from their growth solution by centrifugation at 2500 g for 30 min, and then redispersed in MilliQ water, phosphate-buffered saline (PBS, pH 7.4), HEPES-buffered saline (20 mM HEPES, 150 mM NaCl, pH 7.5), or Tris-buffered saline (10 mM Tris, 150 mM NaCl, pH 7.5) solutions for the stability test at room temperature or 37°C. Extinction spectra and TEM images were collected to monitor the

sample stability. Polystyrene cuvettes used for UV-Vis measurements were pre-coated with 1 wt% Tween 20.

Surface attachment of PEG/NTA on AgDNP@Au

AgDNP@Au were redispersed in MilliQ water after centrifugation at 10870 g for 10 min before further surface modification. For each synthesis, 350 µL of nanoparticle diluted to a peak extinction of 1.16, as measured by UV-Vis spectroscopy, were placed in a 1.5 mL Eppendorf tube. Assuming that all Ag was converted to AgDNP during the nanoparticle synthesis, the concentration of the starting suspension is estimated to be 0.10 nM (see **Text S1, Eq. S3-S5**). A total amount of 375 µM ligand mixture containing mPEG-SH (MW 800, “PEG800” or MW 2000 or “PEG2000”) and NTA was given for reaction at room temperature overnight under 250 rpm shaking. For the samples where only PEG800 or PEG2000 was attached, the Eppendorf tube was pre-coated with 1 wt% aq. Tween 20 for 30 min followed by drying under compressed air.

Ni-incubation to form final PEG/NTA-Ni-modified AgDNP@Au "Nanobiosensors"

After separation from the reaction mixture by centrifugation at 10870 g for 10 min, the PEG/NTA-modified nanoparticles were washed twice with 0.1 M Tris buffer containing 0.025 wt% Tween 20. The particles were then incubated in 50 µM $NiCl_2$ for 2.5 h at room temperature under 250 rpm shaking. Afterwards, the particles were washed once with 20 mM Tris buffer containing 0.005 wt% Tween 20.

Protein-protein interaction sensing, near equilibrium and kinetics

The nanobiosensors were redispersed in different saline buffers in BSA pre-coated cuvettes for protein sensing experiments. BSA pre-coating was performed using a 2.5% (w/v) BSA in PBS buffer, shaken thoroughly to touch all surfaces of the cuvettes for 20 min before removal. The coated surfaces were then washed with Tris-buffered saline and MilliQ H_2O, and dried using compressed air.

Desired amounts of protein stock solution were added to the nanoparticle suspensions (see figure captions for detailed amounts in each measurement). For measurements near equilibrium, the extinction spectrum (350 – 750 nm, 0.2 nm intervals) was monitored until no further peak shift was observable. 250 rpm shaking was applied between measurements to facilitate mixing.

For kinetic measurements, a smaller range of the extinction spectrum (± 10 nm from the peak position λ_{max}) was scanned at a 0.1 nm interval. A fit to a Lorentzian function was performed to determine the λ_{max} value. **Fig. S13** shows a representative spectrum and fit, along with very small fit residuals. Before the addition of the 2nd protein, at least 5 consecutive spectra were measured to determine the starting peak position. The standard error was below 0.012 nm (approx. 0.10 meV). Once the 2nd protein was added, the sample was measured 20-30 s/scan for 25 minutes. After the kinetics measurement, the entire spectrum (350-750 nm, 0.2 nm interval) was scanned again to obtain the “final” spectrum.

The observable binding rate k_{obs} was obtained from an exponential fit of the time-dependent change in the spectral peak λ_{max}. The association rate constant k_a and dissociation rate

constant k_d can be derived from the equation $k_{obs} = [P] \cdot k_a + k_d$ and the equilibrium dissociation constant $K_D = k_d / k_a$. [P] is the concentration of the 2nd protein and K_D is determined from the concentration-dependent "binding curve", as it equals the solution concentration of the 2nd protein at which 50% binding is reached.[4] Each binding curve around an inflection point was fitted with the Hill-Waud equation:[5]

$$\Delta\lambda_{\mathrm{LSPR}} = \frac{\Delta\lambda_m [P]^n}{K_h^n + [P]^n} + \text{offset} \qquad \textbf{Eq. S1}$$

where K_h is the apparent dissociation constant, $\Delta\lambda_m$ is the maximum peak shift of $\Delta\lambda_{LSPR}$ of the 100% bound state, and n is the Hill (cooperativity) coefficient. An offset was only applied when analyzing the second inflection point of a concentration series "binding curve".

By introducing the binding fraction $\theta = \Delta\lambda_{LSPR}/\Delta\lambda_m$, the Hill's coefficient can be derived from the slope of the linear fitting of $\log(\frac{\theta}{1-\theta})$ versus log [P] as follows:

$$log(\frac{\theta}{1-\theta}) = n\log[P] - n\log[K_h] \qquad \textbf{Eq. S2}$$

Protein Cloning

The protein sequences of TAD and NCBD are given in **Table S1**. The DNA sequences were optimized according to the codon usage of E. coli and synthesized by Integrated DNA Technologies (Europe). The genes were cloned to the pETM10 vector with a non-cleavable

N-terminal His_6-tag, and pETM11 vector with His_6-tag followed by a tobacco etch virus (TEV) cleavage site (EMBL, G. Stier), respectively, using NcoI and KpnI restriction sites.

PEX5 (1–113), PEX5 (110–230), and PEX14 (16–80) constructs were obtained from previous work.[6] The His-tagged protein samples were made from the proteins expressed with the pETM10 vector, and the non-His-tagged version of the proteins were obtained from pETM11 constructs by following a TEV protease cleavage to remove the His-tag.

Protein sample preparation

The constructs were transformed into *Escherichia coli* (*E. coli*) BL21 (DE3) cells and expressed in lysogeny broth (LB) medium. A single colony was picked randomly and cultured in the medium with 50 μg/ml kanamycin overnight at 37℃. Overnight cultures were grown in the medium at 37°C, diluted 50-fold, and grown until an optical density of 0.4–0.6 at 600 nm was reached. Then, protein expression was induced by adding 0.5 mM Isopropyl β-D-1-thiogalactopyranoside (IPTG). The cultures were continuously incubated at 37°C for 4 hours and then switched to 25°C for another 20 hours. The cells were harvested by centrifugation at 5000 rpm for 20 min at 4℃. To follow the next purification step, the cell pellets were resuspended in the Ni-NTA binding buffer (30 mM Tris/HCl, pH 8.0, 300 mM NaCl, 10 mM Imidazole, 1 mM TCEP) with the addition of 200 μg/mL lysozyme and 10 μg/mL DNase and lysed by pulsed sonication (3 min, 30% power, large probe, Bandelin UW 2200). The lysates were incubated at 4°C for 20 min to digest chromosomes, followed by the addition of solid urea to the concentration of 4 M, a second sonification step as described above, and then centrifugation at 14000 rpm for 60 minutes at 4 °C.

All proteins were purified using Gravity flow Ni-NTA affinity chromatography (Qiagen) which can be described in three steps. First, a binding step where the supernatant of the cell lysate was applied to the column. Second, a wash step (10X column volume with the binding buffer) where endogenous proteins were removed, and a third step where the protein of interest was eluted from the column with 300 mM imidazole in the binding buffer. The elutes for the non-His-tagged protein sample preparation were subsequently further purified after TEV cleavage running a reverse Ni^{2+} column. All samples were finally purified via size exclusion chromatography (Superdex S75, 16/600, GE) in the buffer (25 mM HEPES, pH 7.5, 150 mM NaCl and 1 mM TCEP). Protein concentrations were determined using the Pierce BCA protein assay kit (Thermo Scientific 23225).

Supporting Figures and Tables

Nanoparticle size distribution

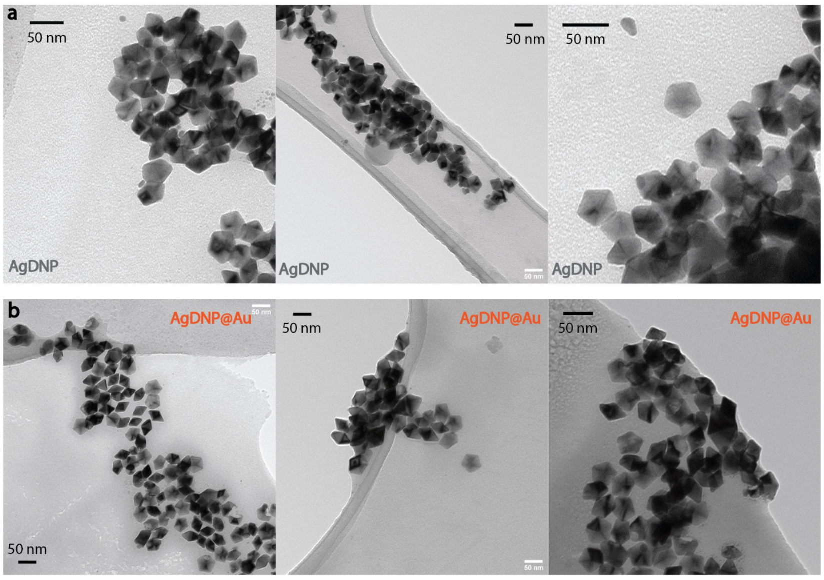

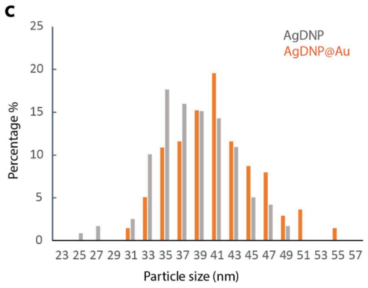

d

	TEM size (nm)	DLS Hydrodynamic size (nm)
AgDNP	38.4 ± 4.5	38.7 ± 1.2
AgDNP@Au	41.0 ± 4.9	42.5 ± 0.7

Figure S1. TEM images of a) AgDNP in post-reaction mixture and b) AgDNP@Au redispersed in 2 mM sodium citrate. Three images are shown for a given sample batch. c) Size distribution of AgDNP and AgDNP@Au obtained from manual analysis of >120 particles in a) and b). d) Mean sizes of AgDNP and AgDNP@Au as determined by TEM and DLS. Although a small number of non-decahedral particles exist, including small spheres, triangular nanoprisms, and some broken decahedral nanoparticles, the vast majority of nanoparticles are intact decahedra. Most rhomboid-like nanoparticles are likely to be decahedral nanoparticles viewed from an orientation where one vertex is hidden.

Thickness of Au coating layer

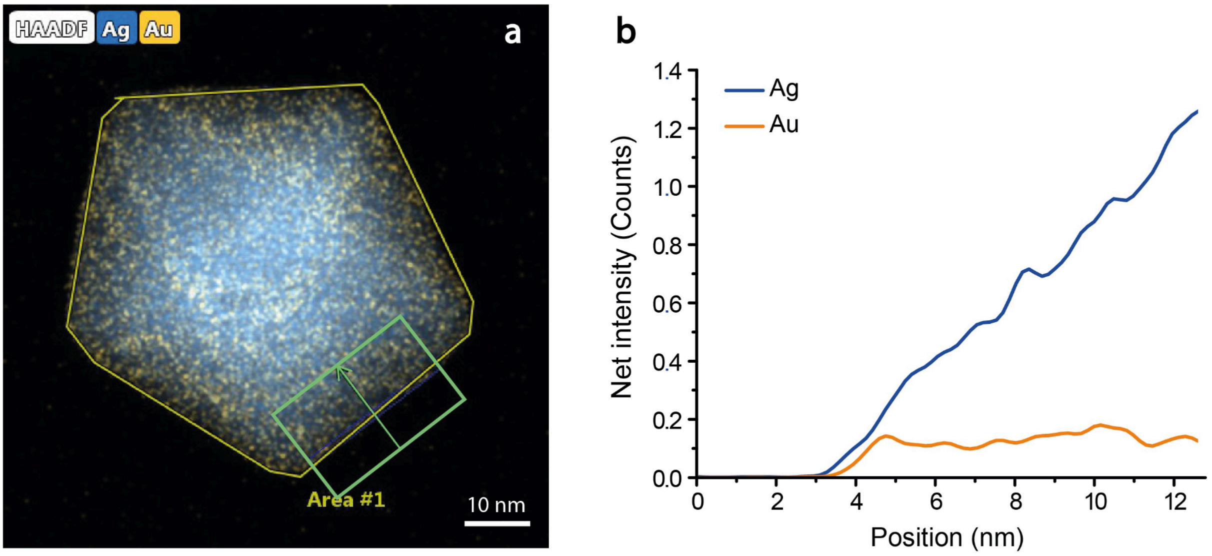

Figure S2. a) STEM-EDX image of a single AgDNP@Au nanoparticle. b) Net intensity of Ag and Au as a function of position. Position 0 starts at the edge of the green rectangle in **a**, and increases along the arrow. The Au coating is roughly 1 nm thick. The formation of Ag-Au alloy cannot be excluded from this result.

Stability of AgDNP with and without Au coating in water and buffer

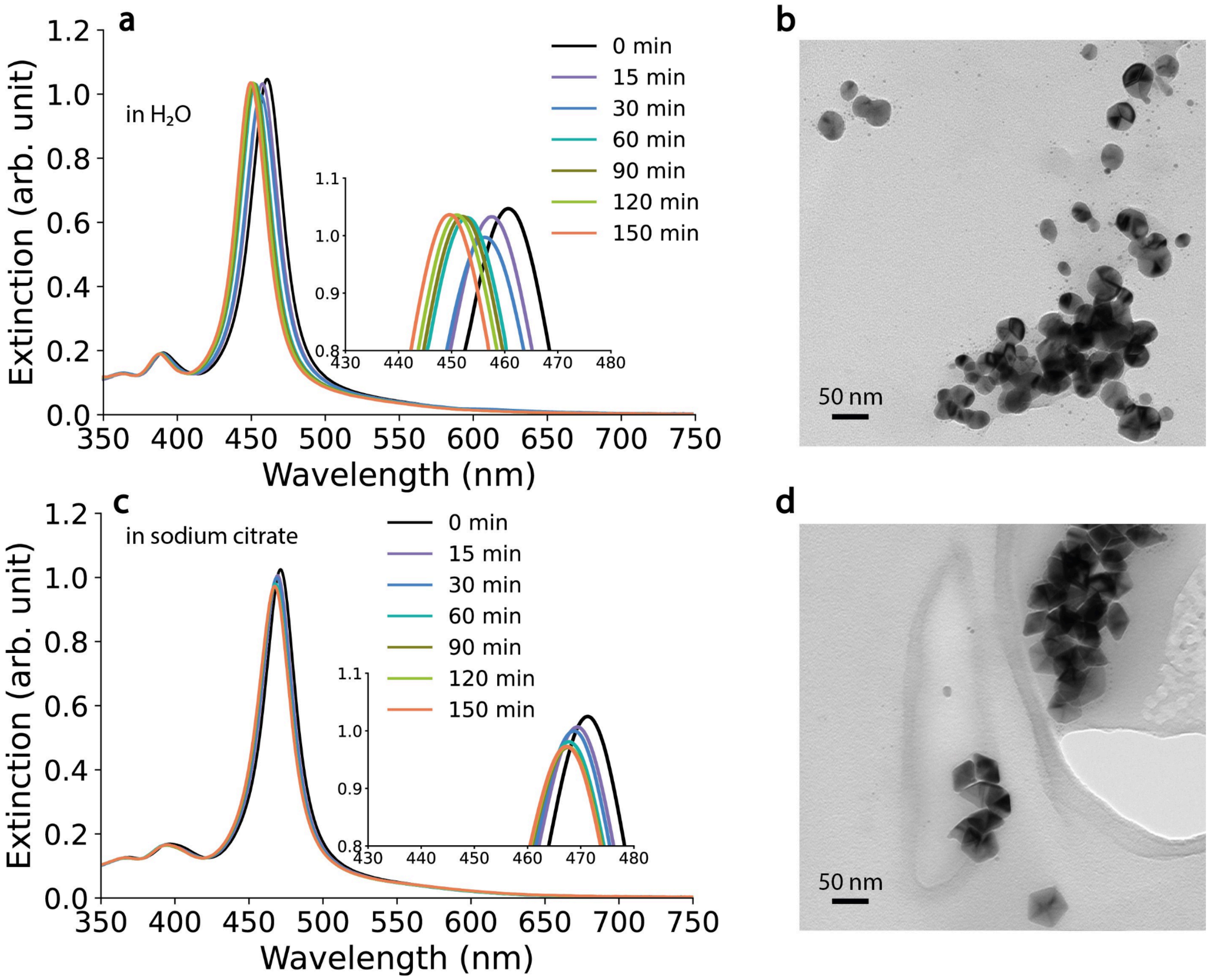

Figure S3. Before Au coating: a) Extinction spectra of AgDNP redispersed in H_2O as a function of time. Insert: zoom-in of the LSPR peak. b) TEM image of AgDNP after redispersion in H_2O for 150 min. The particle tips became rounder and the particle sizes became smaller. c) Extinction spectra of AgDNP redispersed in 2 mM sodium citrate and H_2O as a function of time. Insert: zoom-in of the LSPR peak. d) TEM image of AgDNP after redispersion in sodium citrate for 150 min. The nanoparticles maintained their decahedral shape.

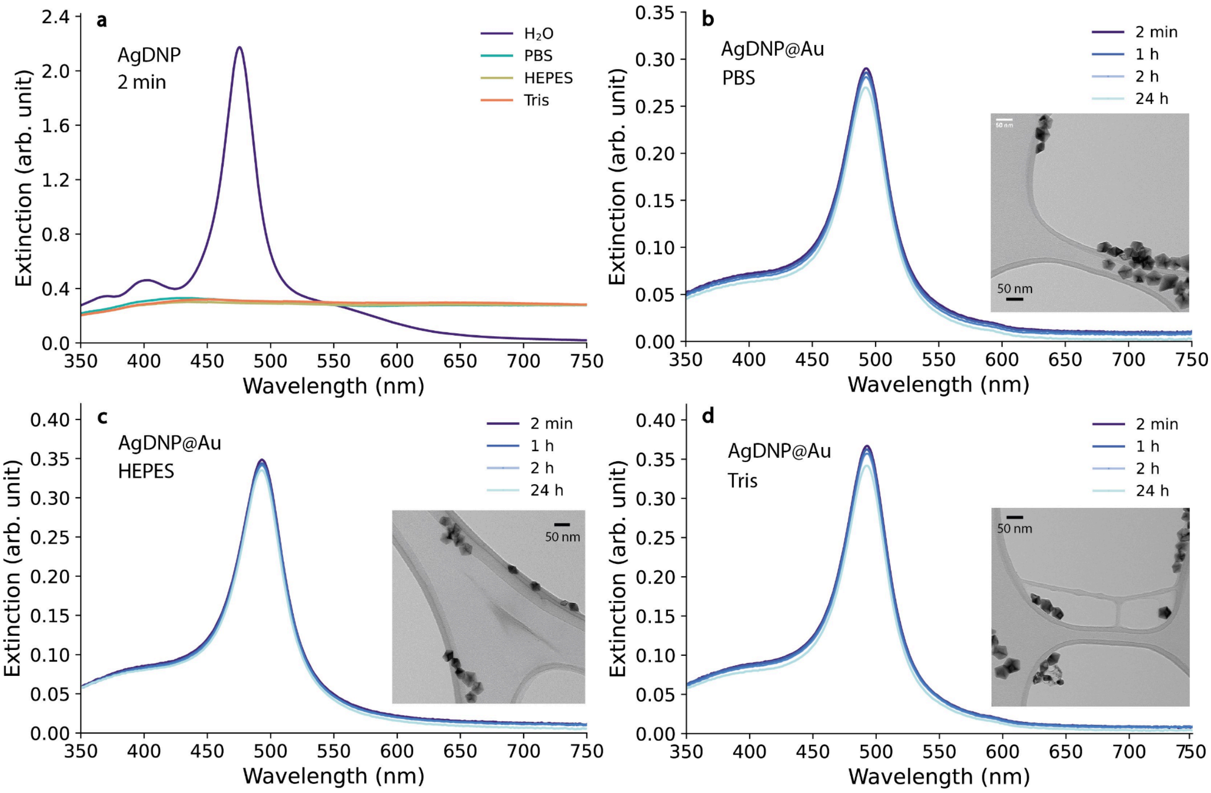

Figure S4. a) Extinction spectra of AgDNP redispersed in H_2O, PBS (pH 7.4), HEPES saline (20 mM HEPES, 150 mM NaCl, pH 7.5), and Tris saline (10 mM Tris, 150 mM NaCl, pH 7.5) after 2 min at room temperature. After Au coating: extinction spectra of AgDNP@Au redispersed in b) PBS (pH 7.4), c) HEPES saline (20 mM HEPES, 150 mM NaCl, pH 7.5), d) Tris saline (10 mM Tris, 150 mM NaCl, pH 7.5) at room temperature at several time points up to 24 h. Insert: TEM images of nanoparticles after storage in the corresponding buffer for 24h, then redispersed in H_2O.

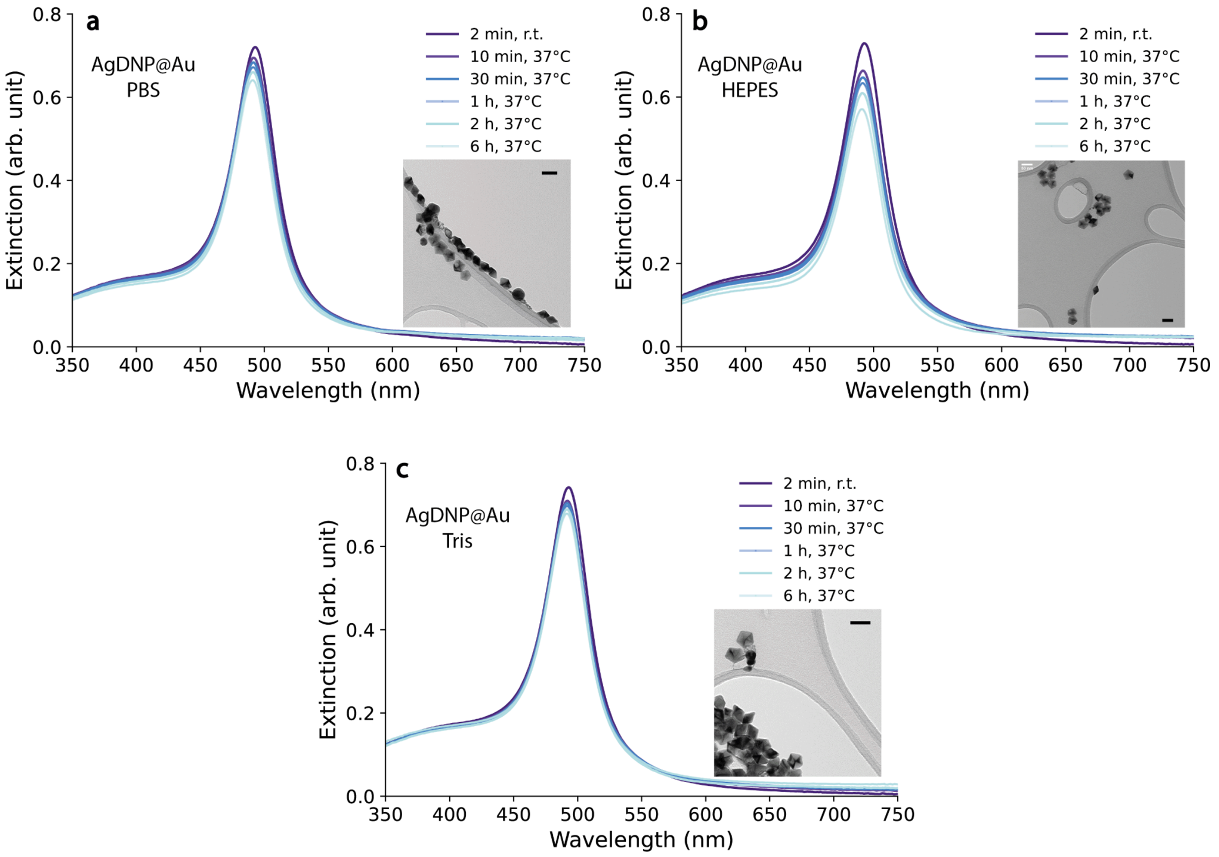

Figure S5. After Au coating at physiological temperature: extinction spectra of AgDNP@Au redispersed in a) PBS (pH 7.4), b) HEPES saline (20 mM HEPES, 150 mM NaCl, pH 7.5), c) Tris saline (10 mM Tris, 150 mM NaCl, pH 7.5) at 37°C for several time points up to 6 h. Insert: TEM images of nanoparticles after storage in the corresponding buffer at 37°C for 6 h, then redispersed in H_2O.

STEM-EELS of PEG/NTA-modified AgDNP@Au

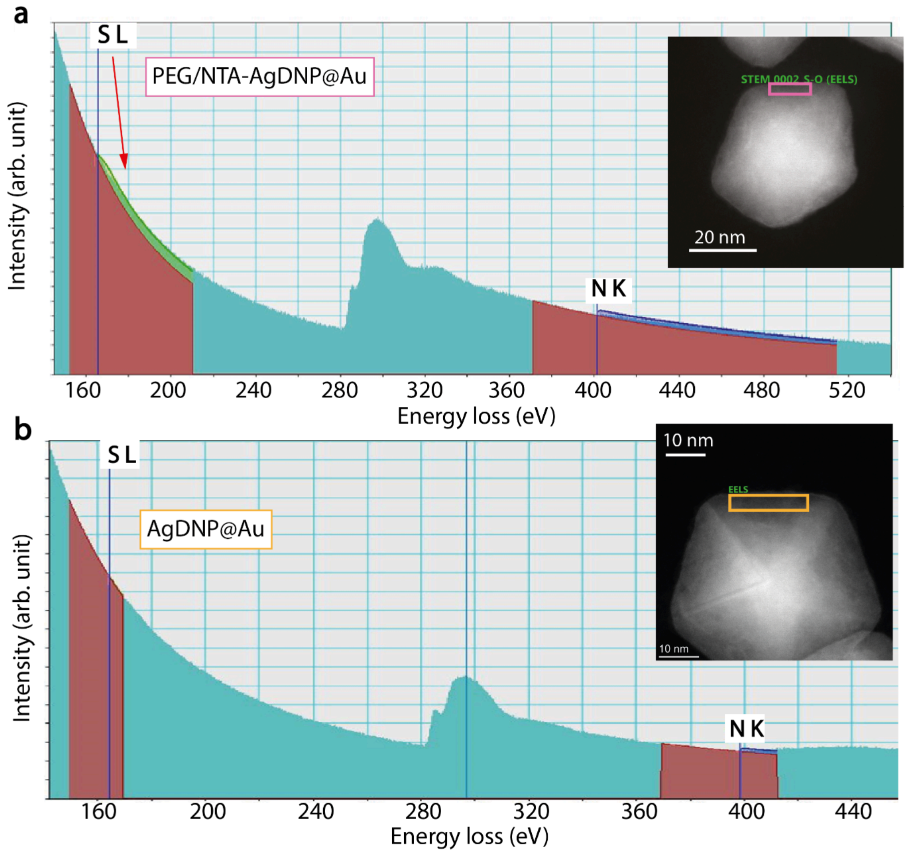

Figure S6. EELS results of a) PEG/NTA-modified AgDNP@Au and b) bare AgDNP@Au confirms the appearance of sulfur after surface modification. The elemental composition of the highlighted areas were measured. The sulfur/nitrogen atomic ratio increases from 0.0044 to 0.129 after PEG/NTA attachment. The small N signal detected on the bare AgDNP@Au likely originates from residual PVP on the particle surface. 80% NTA is shown for consistency with **Figure 2f**.

DLS of AgDNP@Au before and after PEG/NTA-Ni

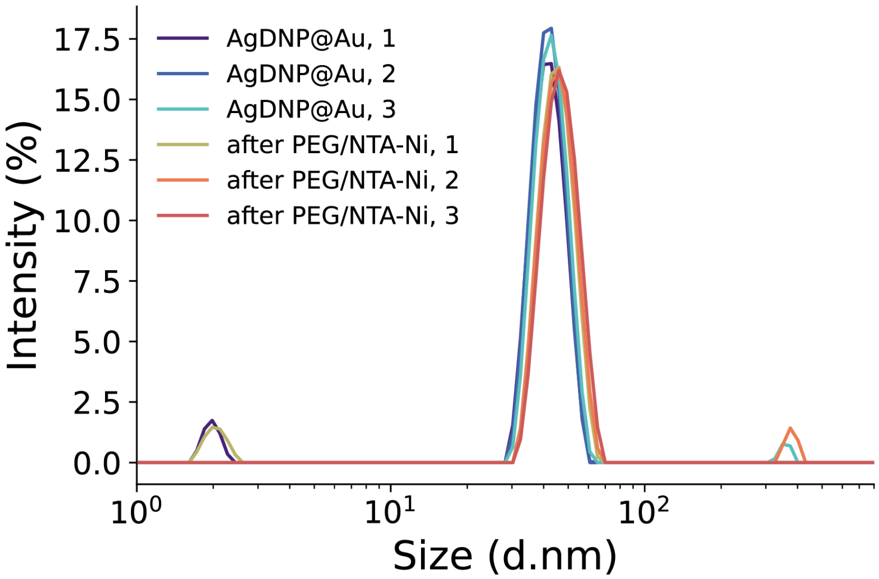

Figure S7. Hydrodynamic size distribution by intensity of Au-coated Ag decahedral nanoparticles measured using DLS before and after two-step PEG800/40%NTA-Ni modification. Measured hydrodynamic sizes were: before modification: 42.5±0.7 nm, after modification: 45.6±1.3 nm.

STEM-EDX of PEG/NTA-Ni-modified AgDNP@Au

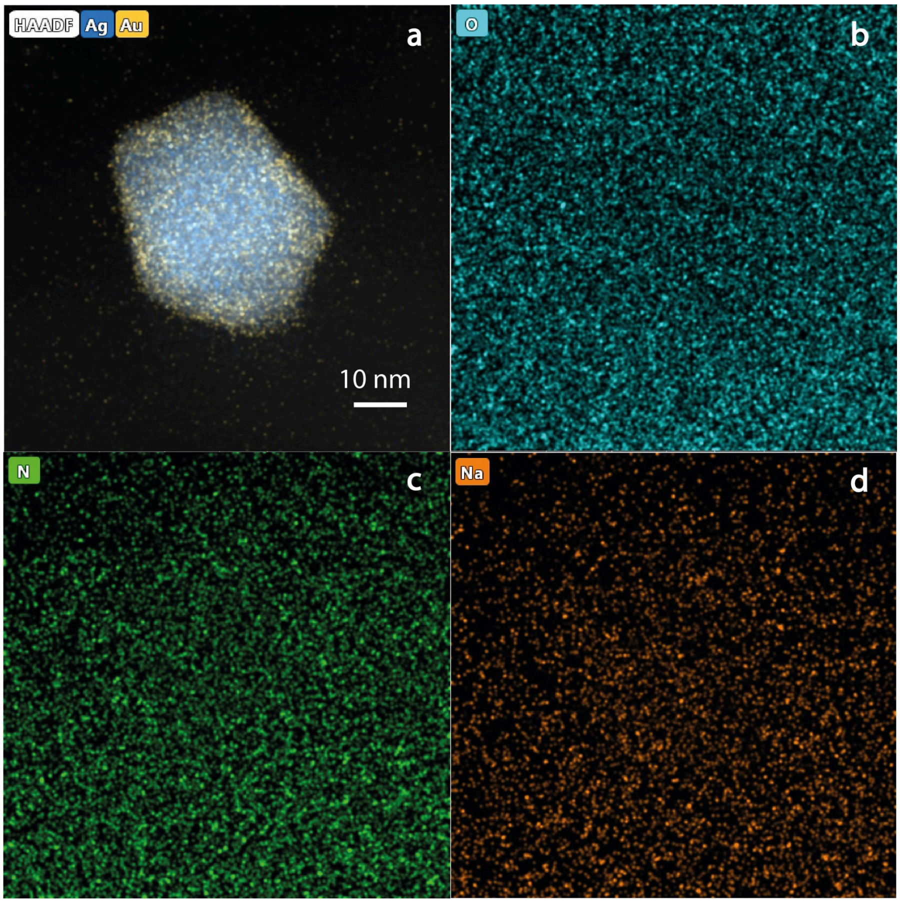

Figure S8. STEM-EDX of PEG/NTA-Ni-modified AgDNP@Au. The scale bar applies to all four channels. a) The overlap of HAADF, Ag, and Au channels. b) O channel. c) N channel. d) Na channel. The O, N, and Na signals in the background around the nanoparticle likely originated from the HEPES buffer, which contains Na^+. In comparison, the Ni channel EDX image (**Fig. 2g**) clearly showed the presence of Ni located on the particle surface due to Ni(II) conjugation to surface NTA.

Sensing the PEX5-PEX14 interaction

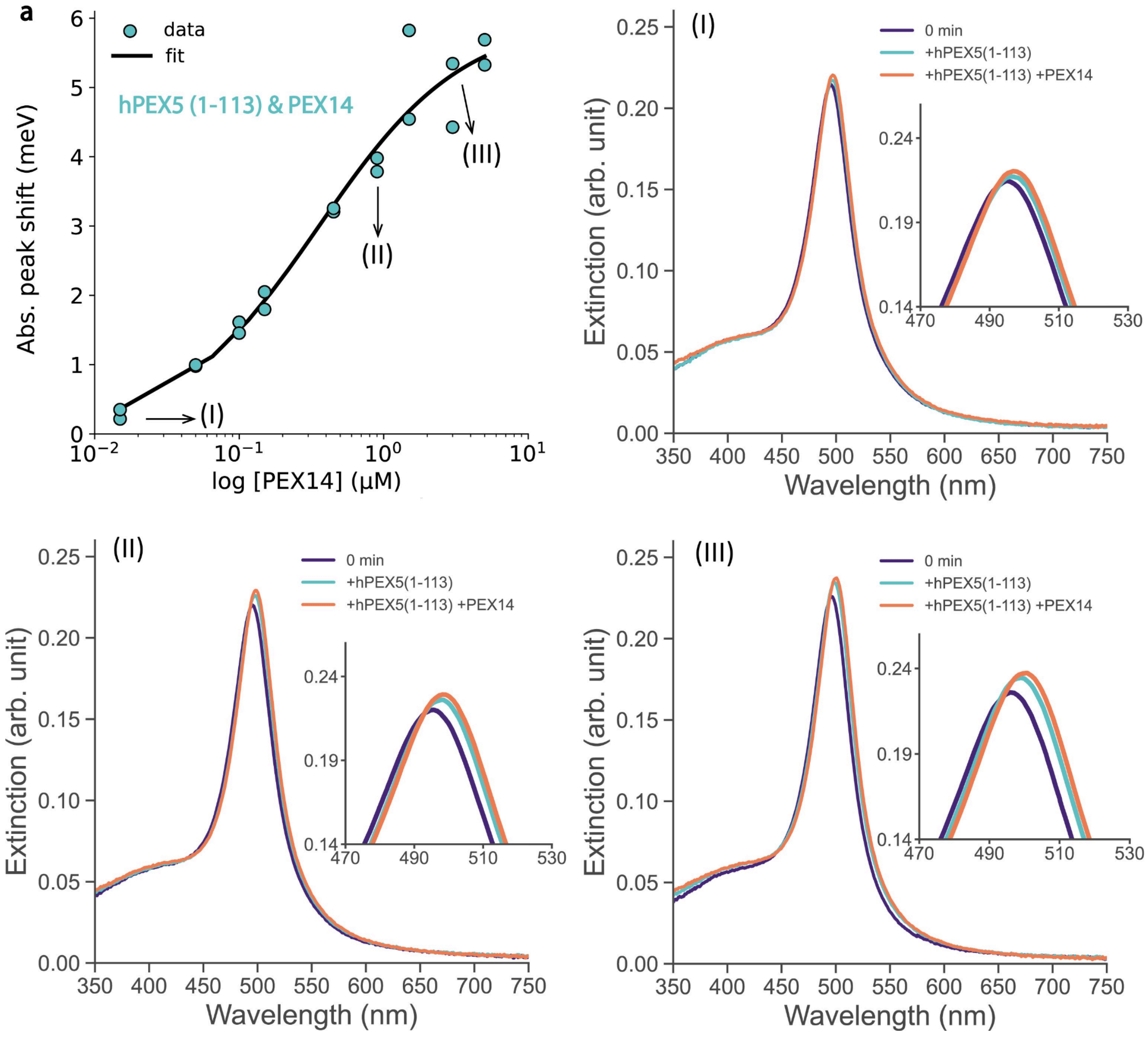

Figure S9. Nanobiosensors maintain colloidal stability over a wide range of PEX14:hPEX5 (1–113) concentration ratio from increasing PEX14 concentration. a) The binding curve of PEX5 (1–113) & PEX14 (16–80) pair, the same as shown in **Fig. 4b**. Selected spectra, and accompanying zoom-in, at (I) PEX14:PEX5 = 1:1, (II) PEX14:PEX5 = 60, (III) PEX14:PEX5 = 200. For each measurement, 0.015 µM hPEX5 construct was added to approx. 0.025 nM nanobiosensors in a 20 mM sodium phosphate buffer containing 100 mM NaCl and 0.025wt% Tween 20 at pH 7.9. The dilution effect caused by the addition of protein solution was corrected.

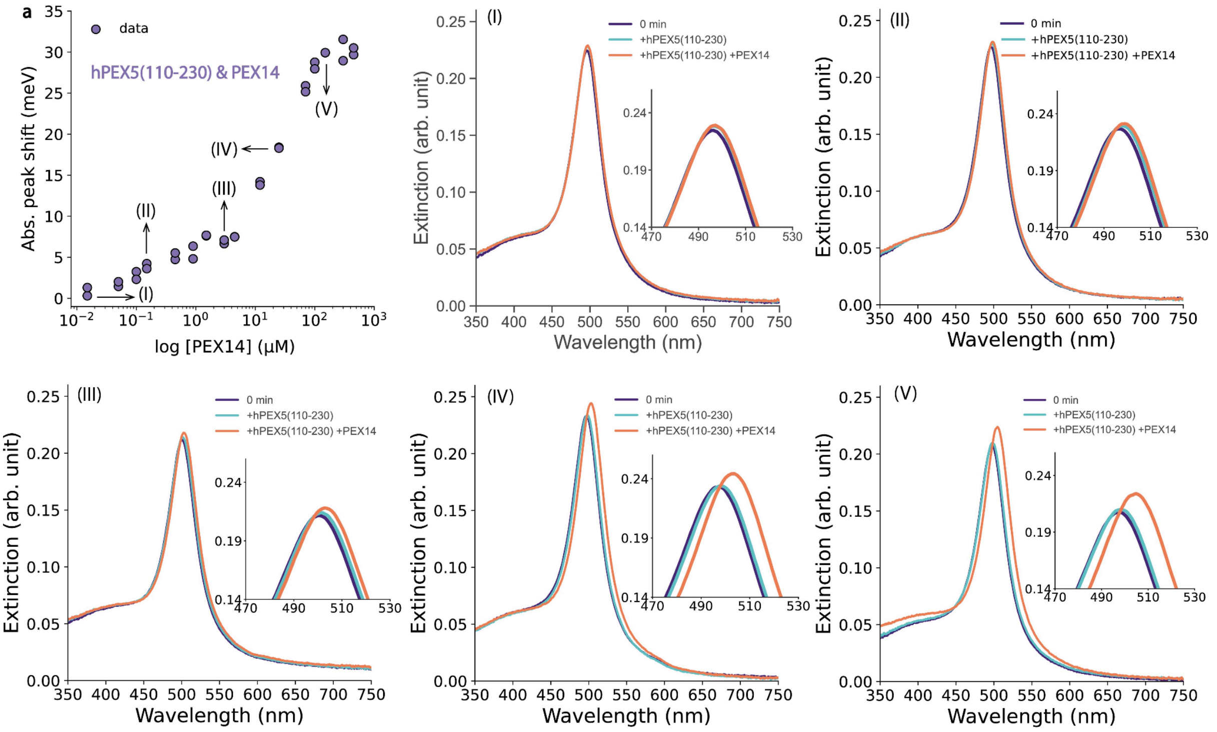

Figure S10. Nanobiosensors remain colloidally stable over a wide range of PEX14:hPEX5 (110–230) concentrations from increasing PEX14 concentration. a) The binding curve of PEX5 (110–230) & PEX14 (16–80) pair, the same as shown in **Fig. 4c**. Selected spectra and zoom-ins of peaks for concentration ratios of (I) PEX14:PEX5 = 1:1, (II) PEX14:PEX5 = 10:1, (III) PEX14:PEX5 = 200:1, (IV) PEX14:PEX5 = 1667:1, (V) PEX14:PEX5 = 10000:1. For each measurement, 0.015 μM hPEX5 construct was added to approx. 0.025 nM nanobiosensors in a 20 mM sodium phosphate buffer containing 100 mM NaCl and 0.025wt% Tween 20 at pH 7.9. The dilution effect caused by the addition of protein stock solution was corrected. Note: although the protein addition at (V) evidently changed the baseline at low wavelengths, it did not cause any significant asymmetric effect around the LSPR peak (± 10 nm).

a

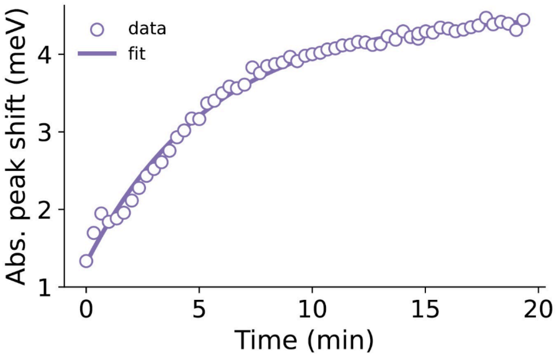

b

hPEX5	PEX14	Binding parameters
110-230	16-80	K_{D1} = 171 nM<br>k_a = 1.24 x 10^4 $M^{-1}s^{-1}$<br>k_d = 2.12 x 10^{-3} s^{-1}<br><br>K_{D2} = 24.7 μM

Figure S11. a) A typical kinetic trace of h-PEX5 (110–230) and PEX14 (16–80) binding at a PEX14:PEX5 ratio of 10:1 without pre-mixing (near the first inflection point in **Fig. 4c**, yellow-squared part). b) Binding parameters of PEX5 (110–230) and PEX14 (16–80) pair. K_{D1} was used to calculate the binding kinetics parameters. Fitting with an exponential function gives an average k_{obs} = 0.00397 s^{-1} from three measurements. 0.015 μM His-Tagged PEX5 construct was added to approx. 0.025 nM nanobiosensors in a 20 mM sodium phosphate buffer containing 100 mM NaCl and 0.025wt% Tween 20 at pH 7.9.

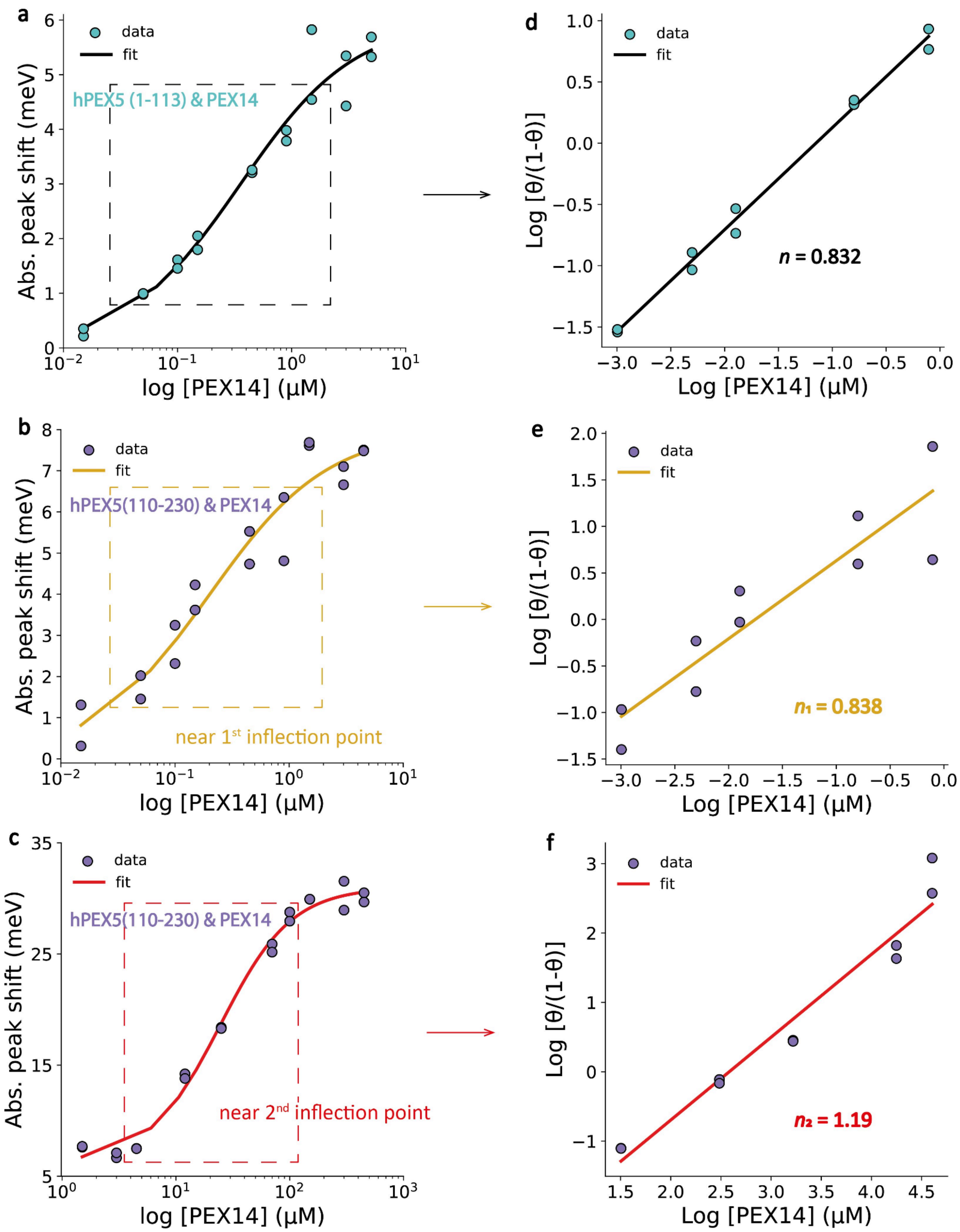

Figure S12. Hill's coefficients (**Eq. S1, S2**) derived from different PEX5-PEX14 binding pairs. a) The binding curve of PEX5 (1–113) and PEX14 (16–80) pair. b) The low-concentration part and c) the high-concentration part of the full binding curve of PEX5 (110–230) and PEX14 (16–80) pair (data same as **Figure 4** of the main text). The corresponding linear fittings using Hill-Waud equation (**Eq. S2**) are shown in d-f).

Fitting the LSPR spectral peak with Lorentzian function

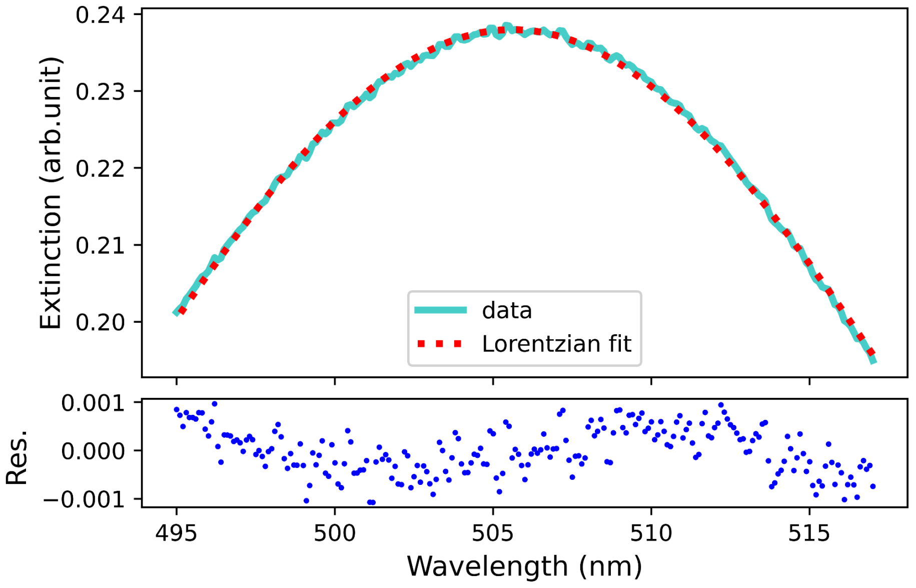

Figure S13. A typical extinction spectrum scanned for kinetic measurements. A fit to a Lorentzian function was performed to derive the λ_{max} value. Residues < 0.001 (arb. unit).

Protein information

Table S1. Selected information on sample proteins.

Protein name	Molar mass (kDa)	Description	Amino acid sequence
His_6-tagged TAD	7.0	Transactivation domain of the tumor suppressor protein p53	MKHHHHHHPMEPLSQETFSDLWKLLPENNVL SPLPSQAMDDLMLSPDDIEQWFTEDPGPD
NCBD	6.9	Nuclear coactivator binding domain of CREB-binding protein (CBP)	GAMEPPRSISPSALQDLLRTLKSPSSPQQQQQV LNILKSNPQLMAAFIKQRTAKYVANQPGMQ
His_6-tagged PEX5 (1-113)	13.3	N-terminal domain of cytosolic peroxisomal targeting protein	MKHHHHHHPMAMRELVEAECGGANPLMKLA GHFTQDKALRQEGLRPGPWPPGAPASEAASKP LGVASEDELVAEFLQDQNAPLVSRAPQTFKMD DLLAEMQQIEQSNFRQAPQRAPGVADLA
His_6-tagged PEX5 (110-230)	15.1	N-terminal domain of cytosolic peroxisomal targeting protein	MKHHHHHHPMADLALSENWAQEFLAAGDAV DVTQDYNETDWSQEFISEVTDPLSVSPARWAEE YLEQSEEKLWLGEPEGTATDRWYDEYHPEEDL QHTASDFVAKVDDPKLANSEFLKFVRQIGEGQ VSLE
PEX14 (16-80)	7.6	N-terminal domain of peroxisomal membrane-associated import protein	GAMATPGSENVLPREPLIATAVKFLQNSRVRQS PLATRRAFLKKKGLTDEEIDMAFQQSGTAADE PSSLW

Supporting Text

Text S1: Estimation of nanoparticle concentration

A rough estimation of maximal AgDNP@Au particle concentration can be made assuming a full conversion of $AgNO_3$ to AgDNP in the AgDNP synthesis. The edge length L_{AgDNP} of Ag decahedral nanoparticles is around 25.0 nm according to TEM images (**Fig. 1b**, **Fig. S1**). To estimate the AgDNP volume, we assume the particle to be in the form of a pentagonal bipyramid with all faces being equilateral triangles. Thus, the volume of each AgDNP is around $9.42\text{x}10^{-18}$ cm^3 as calculated by **Eq. S3**:

$$V_{\text{AgDNP}} = 0.603 \cdot L_{\text{AgDNP}}^3 \qquad \textbf{Eq. S3}$$

The molar mass M_{AgDNP} of AgDNP can be derived from **Eq. S4**, in which d_{Ag} is the density of silver (10.49 g/cm^3), and N_A is the Avogadro constant. The M_{AgDNP} is estimated to be $5.95\text{x}10^7$ g/mol.

$$M_{\text{AgDNP}} = V_{\text{AgDNP}} \cdot d_{\text{Ag}} \cdot N_A \qquad \textbf{Eq. S4}$$

For each AgDNP batch synthesis, the total volume V_{total} of reaction mixture is 15.463 mL (see Method, "Silver decahedral nanoparticle synthesis") in which 0.4 mL, 5 mM $AgNO_3$ is added. The AgDNP concentration c_{AgDNP} is thus 0.235 nM, as calculated by **Eq. S5**, in which M_{Ag} and m_{Ag} are the molar mass and the mass of Ag.

$$c_{\text{AgDNP}} = (m_{\text{Ag}}/M_{\text{AgDNP}})/V_{\text{tot}} = (V_{\text{AgNO3}} \cdot c_{\text{AgNO3}} \cdot M_{\text{Ag}}/M_{\text{AgDNP}})/V_{\text{tot}} \qquad \textbf{Eq. S5}$$

For the synthesis of each AgDNP@Au batch, 3 mL $HAuCl_4$ was slowly added to 3 mL freshly prepared AgDNP reaction mixture (see Method, "Gold coating of silver decahedral nanoparticles"). This means that the concentration of nanoparticles is diluted by half. Hence, assuming all Ag decahedral nanoparticles are coated with Au uniformly, the concentration of AgDNP@Au becomes 0.117 nM, typically aligning with a sample extinction of 1.1 measured by UV-Vis spectroscopy. For each sample batch prepared for surface modification with PEG/NTA, we kept the extinction of the starting AgDNP@Au the same at 1.16 (see Method, "Surface attachment of PEG/NTA on AgDNP@Au"), which roughly corresponds to a nanoparticle concentration of 0.1 nM.

Text S2: Estimation of maximal surface ligand loading

We estimated the maximum possible amount of ligands on the nanoparticle surface by assuming a monolayer attachment, i.e. the maximal ligand loading. To estimate the AgDNP@Au surface area $A_{\mathrm{AgDNP@Au}}$, we assume the particle to be in the form of a pentagonal bipyramid with all faces being equilateral triangles, and

$$A_{\mathrm{AgDNP@Au}} = 4.33 \cdot L_{\mathrm{AgDNP@Au}}^2 \qquad \textbf{(Eq. S6)}$$

As the edge length $L_{\mathrm{AgDNP@Au}}$ of Ag decahedral nanoparticles is around 26.5 nm according to TEM images (**Fig. 1b**, **Fig. S1**), AgDNP@Au surface area $A_{\mathrm{AgDNP@Au}}$ is calculated to be 3.04×10^3 nm^2.

In the presence of high ligand concentrations, ligands can pack densely on the particle surface to achieve a so-called "brush" conformation. In this case, the distance D between two PEG grafts becomes smaller than the Flory radius R_F of the polymer in solution. Li et al. reported a D = 0.7 nm for a dense brush conformation of isocyanate-mPEG (MW= 2000) on ovalbumin nanocarriers.[7] By assuming the same D for mPEG-SH (MW800) and neglecting the size of the PEG molecule itself, the maximal surface ligand loading for our nanoparticles can be estimated to be $A_{AgDNP@Au}/(2D \cdot D) \cdot 2$ = 6206 ligands/particle, i.e. a ligand density of 2.04 chains/nm^2. If true, this would be among the highest PEG graft densities reported.[7–10]

Xue et al. points out that a dense brush conformation is preferably reached at high salt concentrations, and that most PEG brushes are in the so-called ''semidilute'' dense polymer brush (SDPB) regime without becoming more densely packed due to steric hindrance.[8] The SDPB regime is favored for resisting protein adsorption without causing protein unfolding, as this conformation provides a high PEG density while maintaining good chain flexibility. In their work, a high mPEG-SH (MW=2000 kDa) ligand density on 40 nm gold nanospheres at 1.93 chains/nm^2 was achieved in 0.50 M aq. Na_2SO_4 when at least 10^4 eq. PEG molecules were added for nanoparticle surface modification.

In our protocol, we applied 375 μM PEG/NTA ligands to roughly 0.1 nM AgDNP@Au (3.75x10^6 eq., **Fig. 2b**). This is a large excess that should fully load the particle surface, even when the PEG/NTA ligands are arranged in a dense brush form. In the next step, 50 μM $NiCl_2$ was used to saturate the NTA terminal groups with Ni(II) ions.

References

(1) Murshid, N.; Keogh, D.; Kitaev, V. Optimized Synthetic Protocols for Preparation of Versatile Plasmonic Platform Based on Silver Nanoparticles with Pentagonal Symmetries. *Part. Part. Syst. Charact.* **2014**, *31* (2), 178–189.

(2) Murshid, N.; Gourevich, I.; Coombs, N.; Kitaev, V. Gold Plating of Silver Nanoparticles for Superior Stability and Preserved Plasmonic and Sensing Properties. *Chem. Commun.* **2013**, *49* (97), 11355–11357.

(3) Sherry, L. J.; Chang, S.-H.; Schatz, G. C.; Van Duyne, R. P.; Wiley, B. J.; Xia, Y. Localized Surface Plasmon Resonance Spectroscopy of Single Silver Nanocubes. *Nano Lett.* **2005**, *5* (10), 2034–2038.

(4) Jarmoskaite, I.; AlSadhan, I.; Vaidyanathan, P. P.; Herschlag, D. How to Measure and Evaluate Binding Affinities. *Elife* **2020**, *9*. https://doi.org/10.7554/eLife.57264.

(5) Shi, J.; Yang, T.; Kataoka, S.; Zhang, Y.; Diaz, A. J.; Cremer, P. S. GM1 Clustering Inhibits Cholera Toxin Binding in Supported Phospholipid Membranes. *J. Am. Chem. Soc.* **2007**, *129* (18), 5954–5961.

(6) Gaussmann, S.; Gopalswamy, M.; Eberhardt, M.; Reuter, M.; Zou, P.; Schliebs, W.; Erdmann, R.; Sattler, M. Membrane Interactions of the Peroxisomal Proteins PEX5 and PEX14. *Front Cell Dev Biol* **2021**, *9*, 651449.

(7) Li, M.; Jiang, S.; Simon, J.; Paßlick, D.; Frey, M.-L.; Wagner, M.; Mailänder, V.; Crespy, D.; Landfester, K. Brush Conformation of Polyethylene Glycol Determines the Stealth Effect of Nanocarriers in the Low Protein Adsorption Regime. *Nano Lett.* **2021**, *21* (4), 1591–1598.

(8) Xue, Y.; Liu, S.; An, Z.; Li, J.-X.; Zhang, N.-N.; Wang, C.-Y.; Wang, X.; Sun, T.; Liu, K. θ-Solvent-Mediated Double-Shell Polyethylene Glycol Brushes on Nanoparticles for Improved Stealth Properties and Delivery Efficiency. *J. Phys. Chem. Lett.* **2021**, *12* (22), 5363–5370.

(9) Xia, X.; Yang, M.; Wang, Y.; Zheng, Y.; Li, Q.; Chen, J.; Xia, Y. Quantifying the Coverage Density of Poly(ethylene Glycol) Chains on the Surface of Gold Nanostructures. *ACS Nano* **2012**, *6* (1), 512–522.

(10) Lu, J.; Xue, Y.; Shi, R.; Kang, J.; Zhao, C.-Y.; Zhang, N.-N.; Wang, C.-Y.; Lu, Z.-Y.; Liu, K. A Non-Sacrificial Method for the Quantification of Poly(ethylene Glycol) Grafting Density on Gold Nanoparticles for Applications in Nanomedicine. *Chem. Sci.* **2019**, *10* (7), 2067–2074.
